# Untethered thin-film neurostimulator wrapped around tiny nerve trunks for wireless neuromodulation

**DOI:** 10.1126/sciadv.aec9247

**Published:** 2026-06-17

**Authors:** Renyuan Sun, Wenyuan Wang, Wenliang Liu, Ming Yang, Dingke Zhang, Zhonghao Zou, Kun Yang, Chuan Gao, Jiahui She, Ziyu Zhang, Zhi Hu, Feng Hu, Wei Jiang, Kai Shu, Mingxing Xie, Zhouping Tang, Li Zhang, Cunjiang Yu, Zhiqiang Luo

**Affiliations:** ^1^ National Engineering Research Center for Nanomedicine, Research Center for Intelligent Fiber Devices and Equipment, State Key Laboratory of New Textile Materials and Advanced Processing, College of Life Science and Technology, Huazhong University of Science and Technology, Wuhan 430074, China.; ^2^ Department of Ultrasound Medicine, Union Hospital, Tongji Medical College, Huazhong University of Science and Technology, Wuhan 430022, China.; ^3^ Materials Research Laboratory, University of Illinois Urbana-Champaign, Urbana, IL 61801, USA.; ^4^ Brain-Computer Interface Research Institute, Tongji Hospital, Tongji Medical College, Huazhong University of Science and Technology, Wuhan 430030, China.; ^5^ Department of Electrical and Computer Engineering, Department of Materials Science and Engineering, Department of Mechanical Science and Engineering, Department of Bioengineering, Beckman Institute for Advanced Science and Technology, Nick Holonyak Micro and Nanotechnology Laboratory, University of Illinois Urbana-Champaign, Urbana, IL 61801, USA.

## Abstract

Electrical stimulation of tiny peripheral nerve trunks for near-organ neuromodulation enables precise electroceutical therapy for refractory diseases, but long-term stable neuromodulation of these delicate, fragile nerve trunks remains an engineering challenge. Here, we report the development of NeuroWrap Ultrasonic Stimulator (NWUS), an untethered, self-adhesive thin-film neurostimulator capable of conformally wrapping around tiny nerve trunks. The untethered architecture can help avoid the damage or rupture of tiny nerves due to uncontrollable micromotion of lead wires during bodily movements. The NWUS is an ultrasound-responsive acoustoelectric converter with optimized impedance matching, which enables highly effective wireless electrical stimulation. The electroceutical application of NWUS was further illustrated in chronic vagus nerve stimulation for immunomodulation therapy in a rat model. The wireless neuromodulation therapy of rat experimental autoimmune myocarditis was proven to restore left ventricular function, suppress proinflammatory cytokine expression as well as macrophage infiltration within cardiac tissue, and promote regulatory T cell recruitment along with increased anti-inflammatory cytokine levels.

## Teaser

Untethered thin-film neurostimulator conformally wraps around tiny nerves to achieve long-term stable chronic neuromodulation.

## INTRODUCTION

Bioelectronic medicine offers a nonpharmacological approach for chronic disease management by precisely modulating neural activity through implantable electrical devices (*1*–*4*). A notable application of this strategy is near-organ neuromodulation, which directly manipulates peripheral nerves that innervate internal organs and modulates target organ function by sending precise control signals (*5*–*8*). In addition, this approach facilitates an investigation into the effects and mechanisms of neuromodulation on organ function and associated pathologies (*9*–*11*). For instance, inhibition of bladder-projecting sensory afferents enables modulation of bladder overactivity, while electrical stimulation of the splenic nerve reduces proinflammatory cytokine production via norepinephrine (NE) release (*12*, *13*). Anatomically, the vagus nerve has branches directly innervating the heart, offering prospective therapeutic targets for cardiac conditions (*14*, *15*). Recent studies have demonstrated that vagus nerve stimulation (VNS) exhibits potential in the treatment of heart failure and myocardial infarction by activating the cholinergic anti-inflammatory pathway and suppressing excessive sympathetic activity (*16*, *17*). Nevertheless, the tiny neural branches near the organs exhibit notable anatomical variations and are extremely fine, typically only a few hundred micrometers in diameter, and are always located deep within tissues (*18*, *19*). Consequently, designing a neurostimulator suitable for long-term implantation on these tiny and anatomically variable branches, with the aim of achieving near-organ neuromodulation for the treatment of chronic conditions, presents a considerable engineering challenge for translational medicine.

For the fragile neural interface mounted on the near-organ tiny nerve branch, bodily movements can dislodge leads, and the traction forces may damage or even rupture tiny nerve branches (*20*–*22*). Therefore, it is highly demanded to develop soft wireless neurostimulators that can conformally wrap around delicate neural branches to ensure untethered electrode-nerve contact. Meanwhile, a self-adhesive outer layer is critically important to achieve stable fixation and precise positioning within such an untethered architecture (*23*–*26*). Regarding the construction of self-powered battery-free neurostimulators, the wireless power transfer technology, such as ultrasound-responsive soft acoustoelectric converters, would be an excellent candidate, since the ultrasound can penetrate deep tissues (*27*). Specifically, compared to inductive radiofrequency and magnetoelectric strategies, ultrasound offers superior safety-constrained power delivery due to weak absorption attenuation of mechanical waves in soft tissues (table S1); moreover, its compatibility with ultrathin, flexible materials enables the creation of mechanically compliant interfaces that minimize the tissue-device impedance mismatch inherent to rigid magnetoelectric components (*28*–*31*). Despite notable output gains from material and structural optimization of conventional acoustoelectric converters, impedance mismatch between biological tissue and ultrasound-responsive devices considerably reduces energy transfer efficiency, which is still a technical challenge to be better addressed (*32*–*34*). For instance, a capacitive matching approach had recently been explored to improve the performance of ultrasound-driven triboelectric nanogenerators (TENGs), but these electrical components inevitably increase the overall device size, potentially causing tissue damage and provoking inflammatory responses (*35*). Ideally, an untethered near-organ neurostimulator should fulfill three key criteria: (i) an ultrathin, mechanically compliant architecture that conforms gently to the surfaces of tiny nerve trunks; (ii) a self-adhesive, insulating encapsulation layer that provides stable and secure nerve contact without the need for mechanical suturing; and (iii) high-performance miniature acoustoelectric converters with optimized impedance matching for highly effective neurostimulation.

**Fig. 1.**
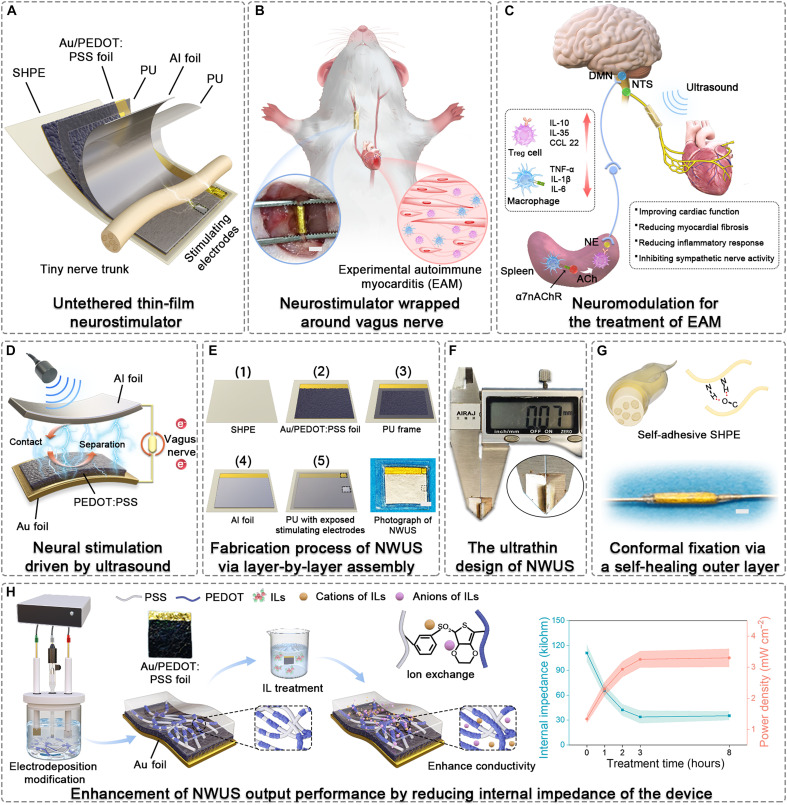
Wireless neuromodulation by untethered thin-film neurostimulator. (**A**) Schematic of the NWUS for neural stimulation. (**B**) Schematic of ultrasound-driven electrical stimulation using NWUS wrapped around rat vagus nerve. Scale bar, 3 mm. (**C**) Schematic of NWUS-enabled VNS for treating EAM. (**D**) The mechanism of ultrasound-driven triboelectric power generation in NWUS. (**E**) Schematic of the layer-by-layer assembly of NWUS using five thin-film layers. Scale bar, 2 mm. (**F**) Photograph demonstrating the ultrathin form factor of the NWUS. (**G**) Conformal fixation of NWUS on the tiny nerve trunks by the self-healing properties of SHPE. Scale bar, 1 mm. (**H**) Electrochemical deposition of PEDOT:PSS on Au foil followed by IL treatment, which reduced the internal impedance of NWUS and subsequently increased its output power density. DMN, dorsal motor nucleus; NTS, nucleus tractus solitarius; α7nAChR, α7 nicotinic acetylcholine receptor; ACh, acetylcholine; NE, norepinephrine.

Here, we introduce an untethered thin-film neurostimulator capable of conformally wrapping around tiny nerve trunks, achieving long-term and stable neuromodulation. The NeuroWrap Ultrasonic Stimulator (NWUS) is constructed from five flexible ultrathin layers and includes exposed stimulation electrodes for direct contact with nerve tissue (Fig. 1A). As shown in Fig. 1B, NWUS can be conformally wrapped around the rat vagus nerve (diameter of ~250 μm) to deliver electrical pulses for treating experimental autoimmune myocarditis (EAM) in a rat model. Driven by external programmable ultrasound pulses, NWUS-enabled wireless neurostimulation demonstrated its immunomodulation effects and consequent improvement of cardiac function (Fig. 1C). Mechanistically, NWUS uses ultrasound-driven contact separation between its poly(3,4-ethylenedioxythiophene):poly(styrene sulfonate) (PEDOT:PSS) and aluminum (Al) layers to drive charge transfer, converting mechanical energy into electrical charge for neural stimulation (Fig. 1D). To implement this mechanism in an ultrathin and flexible format, the device was fabricated via a straightforward layer-by-layer assembly process (Fig. 1E). As shown in Fig. 1F, the rough thickness of our thin-film device measured by a vernier caliper is about 70 μm, and the precise total thickness of 79.3 μm was quantified using a high-precision multifunction thickness tester. A self-healing polymer elastomer (SHPE) was used as the outermost layer to ensure conformal, stable fixation to nerve tissue while minimizing mechanical stress at the interface (Fig. 1G). To maintain the ultrathin architecture of NWUS, PEDOT:PSS was electrochemically deposited onto gold (Au) foil to form one half of the triboelectric pair (with Al serving as the counterpart). Furthermore, ionic liquid (IL) treatment was applied to the PEDOT:PSS coating to increase the conductivity of PEDOT:PSS thin film. The IL treatment also reduced the internal impedance of the assembled NWUS and helped increase the output power density under a load comparable to that of the vagus nerve (Fig. 1H). Together, these device innovations enable untethered, chronic neural stimulation for targeted neuromodulation, demonstrating its broad potential for wireless electroceutical therapy.

## RESULTS

### Design and fabrication of NWUS

Neuromodulation of microscale peripheral nerves demands an ultrathin, mechanically compliant device capable of long-term stable fixation. To meet these requirements, NWUS uses a five-layer thin-film architecture, as detailed in Fig. 1E and fig. S1. The outermost layer consists of a ~30-μm-thick SHPE, which provides a soft, self-healing substrate for conformal nerve wrapping. NWUS delivers neural stimulation based on the principle of a TENG. To implement this mechanism in an ultrathin and efficient form factor, the second layer comprises a composite foil (~10-μm thick), fabricated by electrochemically depositing PEDOT:PSS onto ultrathin Au foil, which serves as one half of the triboelectric pair (fig. S2, A and B). The complementary half is formed by an Al foil (~15-μm thick) incorporated in the fourth layer. A commercial polyurethane (PU) is positioned between the triboelectric layers to physically separate their surfaces and enable their contact-separation motion under ultrasound driving for charge generation. Last, an adhesive PU film encapsulates the assembly by bonding to the SHPE layer while featuring two 0.6 mm–by–0.6 mm cutouts that expose the Au and Al stimulation electrodes for direct nerve contact. Within this multilayer architecture, the overall device dimensions and electrode spacing were specifically engineered to match the anatomical constraints of the rat cervical vagus nerve (~250 to 300 μm), enabling conformal electrode-nerve contact for efficient acoustoelectric conversion while avoiding excessive mechanical compression or vascular disruption. This ultrathin and mechanically compliant construction allows NWUS to safely conform to microscale nerves and support long-term, stable neuromodulation.

Optimization of the internal impedance of NWUS, aimed at maximizing output power near the neural load, was achieved by electrochemically depositing PEDOT:PSS onto ultrathin Au foil as one component of the triboelectric pair (fig. S3). The electrical performance of the PEDOT:PSS layer can be further enhanced through IL treatment, which facilitates cation-anion interactions where cations preferentially associate with the PSS domains and anions interact with PEDOT chains. These interactions are proposed to weaken the Coulombic attraction between PEDOT and PSS, thereby promoting phase separation (Fig. 1H) (*36*). Consequently, the PEDOT chains are reorganized into a more compact and ordered structure, enhancing the film’s surface smoothness. Atomic force microscopy reveals that the surface peak height decreases from ~100 to 50 nm posttreatment, with a more uniform height distribution (fig. S4, A and B). Electrochemical impedance spectroscopy shows that the Au/PEDOT:PSS foil has lower charge-transfer resistance than bare Au, indicating enhanced electrochemical performance (fig. S5A). This impedance continuously decreased with prolonged IL treatment, dropping from an initial ~110 to ~35 kilohms, until reaching a plateau at around 3 hours. Beyond this point, no further improvement was observed, suggesting that the enhanced interfacial conductivity and ion mobility had reached saturation due to sufficient phase separation and structural reorganization within the PEDOT:PSS layer. Notably, the phase angle at high frequencies (above 100 kHz) increases with longer IL treatment, reflecting a shift in capacitive behavior and improved interfacial properties (fig. S5B).

Further insight into the compositional changes responsible for the impedance improvement was obtained via x-ray photoelectron spectroscopy, which confirmed the chemical states of sulfur in PEDOT and PSS (fig. S6A). By fitting and integrating the S 2p peaks, the relative PEDOT:PSS ratio was quantified. Following IL treatment, the PEDOT fraction gradually increased (fig. S6B). The untreated sample exhibits a PEDOT:PSS ratio of 0.28, which increases to 1.94 after 3 hours of treatment, indicating the formation of more PEDOT-rich domains that contribute to enhanced conductivity (*37*). The IL-treated Au/PEDOT:PSS foil was then integrated into NWUS, and compared to the untreated state, the 3-hour IL treatment markedly reduced the device’s internal impedance to 35 kilohms (Fig. 1H), which closely matches the impedance of neural tissue at frequencies above 500 kHz (fig. S7). Based on these findings, 3-hour IL-treated Au/PEDOT:PSS foil was selected for all subsequent NWUS devices to ensure consistent low internal impedance and high electrical performance. In addition, to evaluate the long-term stability of the foil, it was soaked in 0.1 M phosphate-buffered saline (PBS) for 4 weeks, followed by electrochemical impedance measurements. The results (fig. S8) showed that while the high-frequency intercept remained stable, a slight increase in the semicircle diameter was observed in the Nyquist plots after 4 weeks. This indicates a minor rise in charge-transfer resistance, likely due to slight reorganization of conductive pathways within the PEDOT:PSS network, yet the device retains sufficient conductivity for effective stimulation.

To minimize surgical complexity and eliminate the need for secondary fixation, SHPE is used in NWUS as the outermost encapsulation layer (Fig. 1G). Specifically, SHPE was synthesized using polytetrahydrofuran (PTHF) as soft segments and dimethyl oxime as hard segments. Fourier-transform infrared analysis reveals a peak at 1105 cm^−1^ for ether functional groups and peaks at 1238 and 1732 cm^−1^ for ester functional groups, with additional peaks at 2864 and 3372 cm^−1^ corresponding to ─CH_2_─ and N─H stretching vibrations (fig. S9). Nuclear magnetic resonance analysis corroborates these assignments, confirming the successful incorporation of the designed soft and hard segments (fig. S10). The PTHF soft segments reduce crystallinity and impart high stretchability and biocompatibility, whereas the dimethyl oxime hard segments enhance toughness and self-healing capacity. Mechanically, SHPE endures up to ~950% linear strain and exhibits a tensile modulus of 470 kPa (fig. S11A), reflecting enhanced chain flexibility conferred by the PTHF segments. The shear modulus is 2.03 MPa (fig. S11B), which may result from enhanced chain mobility. To further assess chronic mechanical stability, cyclic tensile testing was performed for 1000 cycles. The SHPE exhibited characteristic viscoelastic hysteresis due to the dynamic hydrogen bonding network, which dissipates energy through bond dissociation and reformation. Crucially, the material maintained structural integrity without fracture (fig. S12). Given the low-strain physiological environment of the vagus nerve, this fatigue resistance ensures robust long-term encapsulation. Peel tests record an adhesion energy of 612 ± 22.63 J m^−2^ after a 2-min healing period (fig. S13A). In addition, SHPE demonstrated rapid self-healing owing to the multiple hydrogen bonds within it. Surface scratches on the SHPE film self-healed at room temperature within 10 min (fig. S14). Notably, structural integrity is maintained by the strong adhesion between the outer SHPE and the inner PU sealing layer. Peeling tests confirmed an interfacial toughness of 427 J m^−2^ between these encapsulation layers (fig. S13B), ensuring excellent mechanical stability. These findings indicate that SHPE affords rapid, complete self-healing, ensuring electrode stability and minimizing mechanical stress on the neural tissue during implantation.

### Electrical performance of ultrasound-driven NWUS

**Fig. 2.**
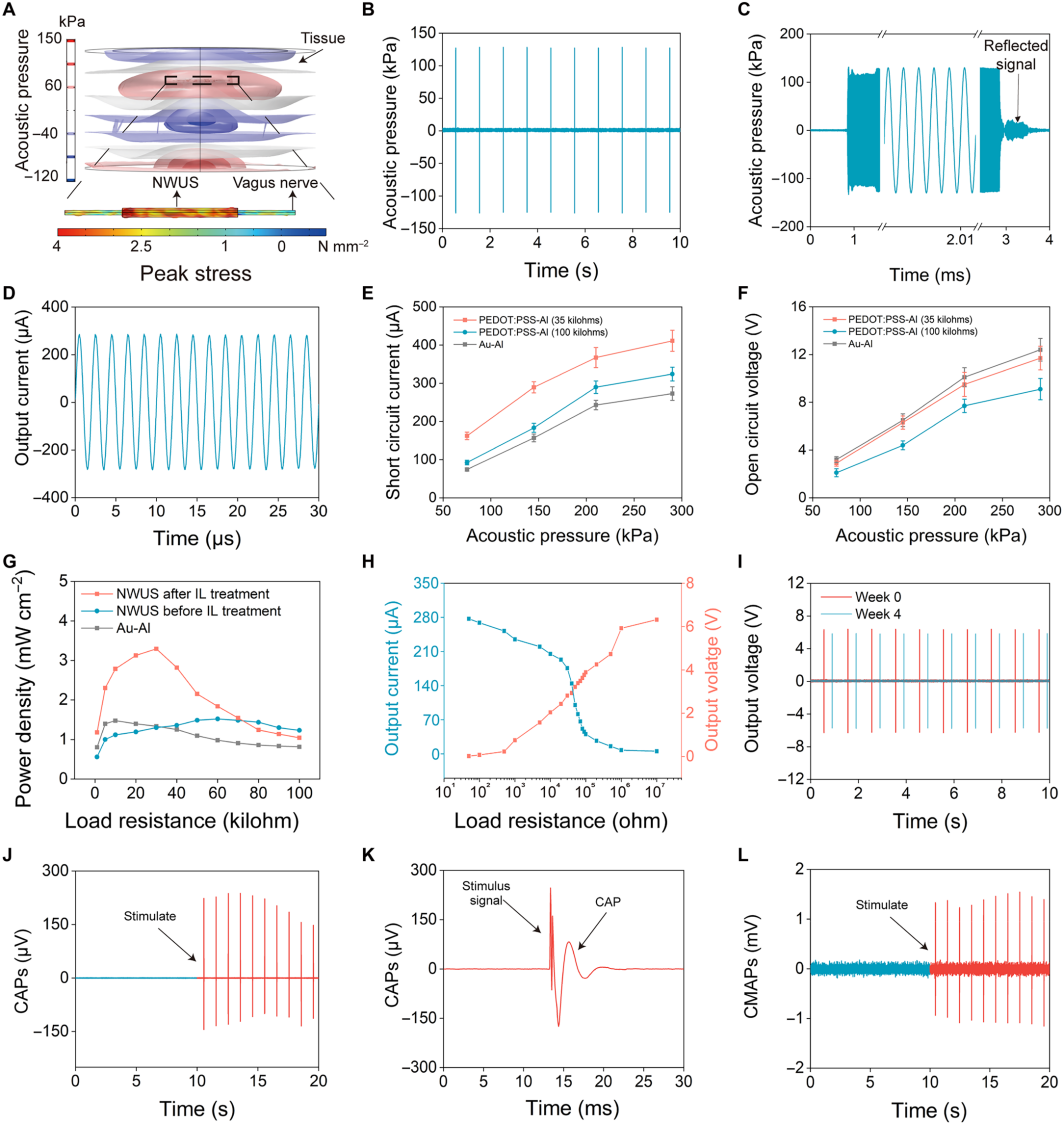
Ultrasound response and electrical output performance of NWUS. (**A**) COMSOL Multiphysics simulation illustrating the acoustic pressure distribution and structural deformation of the NWUS under 150-kPa ultrasound excitation. (**B**) Measurement of the ultrasound acoustic intensity generated by an ultrasound transducer using a hydrophone immersed in water. (**C**) Ultrasonic waveform driven by 500-kHz sine wave, comprising 1000 cycles, with clear acoustic reflection signals. (**D**) The output current generated by NWUS under ultrasound drive (ultrasound frequency, 500 kHz; acoustic pressure, 150 kPa). (**E**) Short circuit current of NWUS before and after IL treatment and of the Au-Al TENG under varying acoustic pressures. (**F**) Open circuit voltage of NWUS before and after IL treatment, and of the Au-Al TENG under varying acoustic pressures. (**G**) Power density of NWUS before and after IL treatment, and of the Au-Al TENG under different load resistances. (**H**) Output voltage and current of NWUS after IL treatment under different load resistances. (**I**) Comparison of the output voltage of NWUS after 4-week immersion in PBS. (**J**) CAPs induced by VNS via ultrasound-driven NWUS. (**K**) Characteristic CAP waveform induced by stimulus signal. (**L**) CMAPs induced by sciatic nerve stimulation via ultrasound-driven NWUS. Data in (E) and (I) are presented as means ± SD from *n* = 5 independent devices.

The feasibility of ultrasound-driven energy transmission was evaluated through COMSOL Multiphysics® (v6.3) simulations under an acoustic excitation of 150 kPa (Fig. 2A), which showed that the combined effects of incident and reflected waves efficiently deliver mechanical energy to NWUS. Safety analysis further demonstrated that the generated acoustic pressure at 150 kPa remains well below the threshold for biological tissue exposure (fig. S15) (*38*). The ultrasound transducer was then driven by a pulsed signal (2-ms width, 1-s interval), and the resulting acoustic intensity in water was quantified using a calibrated hydrophone (Fig. 2B). Each pulse comprised 1000 cycles of a 500-kHz sine wave (Fig. 2C). Then, the relationship between driven voltage and resulting acoustic pressure was characterized (fig. S16), showing a linear increase in measured pressure from 75 ± 5.4 to 281 ± 13.2 kPa as the driven voltage increased from 25 to 100 V. To assess spatial sensitivity, lateral displacement of the hydrophone (0 to 10 mm) revealed a pressure drop to 72.4% at 10 mm off-axis, suggesting moderate tolerance to transducer misalignment (fig. S17). Collectively, these simulation and experimental results demonstrate the transducer enabling stable and programmable acoustic energy delivery.

To mimic in vivo nerve geometry, NWUS was wrapped around a 300-μm-diameter silicone wire and fully immersed in water for electrical performance evaluation (fig. S18). The ultrasound transducer was positioned 5 mm anterior to the NWUS, corresponding to the typical subcutaneous depth of the cervical vagus nerve in rats. Under ultrasound driving, the output current waveform of the NWUS closely matched the input sinusoidal signal (Fig. 2D), indicating reliable acoustic-to-electrical conversion. Notably, the open circuit voltage and short circuit current of the wrapped NWUS reached ~87.6 and 91.9%, respectively, of those measured from the planar NWUS under identical conditions (fig. S19, A and B). The electrical performance of NWUS was systematically measured across acoustic pressures ranging from 75 to 290 kPa, using an Au-Al TENG as control. Both open circuit voltage and short circuit current increased substantially after IL treatment. This enhancement can be attributed to the improved interfacial charge transfer facilitated by the matched density of states between PEDOT:PSS and Al. As a result, the NWUS generated markedly higher short circuit current compared to the Au-Al TENG (Fig. 2, E and F). Consistent with these performance improvements, the NWUS achieves a maximum acoustoelectric conversion efficiency of ~2.22% under an acoustic pressure of 150 kPa, representing a notable improvement over previously reported ultrasound-driven triboelectric devices (*39*, *40*). To verify that the electrical output originates from the triboelectric effect rather than electromagnetic interference, we conducted rigorous shielding experiments: The ultrasound transducer was wrapped in grounded aluminum foil, and all measurement leads were referenced to a common ground. The output signals measured with and without this additional shielding showed no detectable difference (fig. S20), confirming that triboelectric generation at the PEDOT:PSS-Al interface dominates the response. Furthermore, the transferred charge density exhibited a linear relationship with acoustic pressure up to 210 kPa (fig. S21). Beyond this threshold, the charge density plateaued, which may be attributable to surface charge saturation of the device. In addition, the NWUS maintained high energy transfer efficiency under lateral misalignment of up to 10 mm from the acoustic axis (fig. S22), demonstrating broad positional tolerance that accommodates physiological micromotion and minor placement variations during chronic implantation without compromising therapeutic efficacy.

Approximating in vivo electrical loading conditions involved measuring the resistance of the 1-mm-long rat vagus nerve, which ranged from ~18 to 180 kilohms over frequencies spanning 1 kHz to 1 MHz (fig. S7). Based on these values, the power density of NWUS (both before and after IL treatment) and the Au-Al TENG was evaluated across a range of load resistances from 1 to 100 kilohms. The IL-treated NWUS exhibited a peak power density at a 30-kilohms load, closely matching the impedance of a 1-mm-long vagus nerve at 500 kHz, indicating excellent matching for effective energy transfer under physiological conditions. Crucially, NWUS exhibits a broad high-power density window (5 to 50 kilohms) that encompasses the measured physiological impedance of rat sciatic (~10 kilohms) and vagus (~28 kilohms) nerves at 500 kHz, ensuring approximate stimulation efficacy despite individual anatomical variations. Furthermore, the device offers tunable impedance matching via adjustment of electrode spacing, facilitating optimization for diverse neural targets and cross-species applications. As shown in Fig. 2G, the untreated NWUS exhibited a peak power density at 60 kilohms, and the Au-Al TENG peaked at 10 kilohms, underscoring the impedance-matching improvement achieved via IL treatment. The voltage and current output characteristics of NWUS after IL treatment, measured across load resistances ranging from 50 ohms to 10 megohms, are presented in Fig. 2H (the corresponding data for NWUS before IL treatment and the Au-Al TENG are shown in fig. S23, A and B). As the load resistance increased, the output voltage gradually rose and reached a peak, whereas the output current exhibited an inverse trend, decreasing with higher resistance. In addition, the NWUS demonstrated robust stability, retaining over 93.2% of its electrical output after 4 weeks of PBS immersion (Fig. 2I) and maintaining consistent performance over 3 hours of continuous pulsed ultrasound excitation (fig. S24).

### Biocompatibility assessment of NWUS

**Fig. 3.**
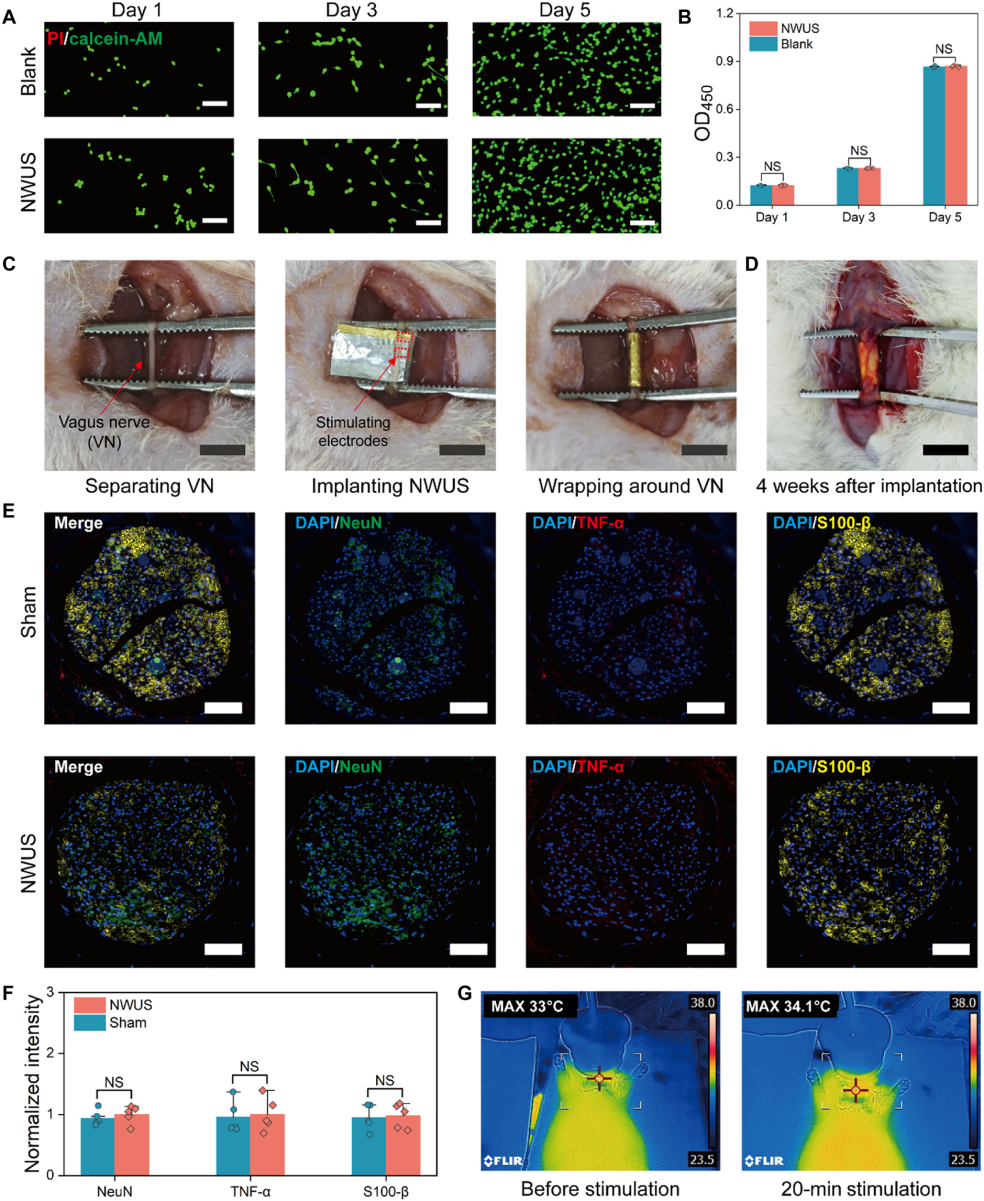
In vitro and in vivo biocompatibility assessment of NWUS. (**A**) Representative live/dead staining images of PC12 cells cocultured with NWUS extract at 1, 3, and 5 days. Scale bar, 100 μm. PI, propidium iodide. (**B**) Viability of PC12 cells cocultured with the NWUS extract at 1, 3, and 5 days (*n* = 5 independent samples). OD_450_, optical density at 450 nm. (**C**) Process of NWUS implantation. Scale bars, 5 mm. (**D**) Photograph of NWUS 4 weeks postimplantation in vivo. Scale bar, 5 mm. (**E**) Representative immunofluorescence staining of the vagus nerve cross-sections 4 weeks postimplantation. Scale bars, 50 μm. (**F**) Normalized fluorescence intensity of NeuN, TNF-α, and S-100β from the NWUS group and sham group (*n* = 5 independent animals). (**G**) Negligible temperature rise after 20-min electrical stimulation by ultrasound-driven NWUS. Data are presented as means ± SD in (B) and (F) and were analyzed by one-way analysis of variance (ANOVA) first and then by Tukey’s post hoc test. NS, not significant.

To comprehensively evaluate the biocompatibility of NWUS, both in vitro and in vivo studies were conducted. In the in vitro experiments, cell counting kit-8 assays and live/dead staining were conducted to investigate the potential cytotoxicity of NWUS. Neural (PC12) cell line and mouse fibroblast (L929) cell line were selected for coculture with NWUS extract over a period of 5 days. Standard Dulbecco’s modified Eagle medium without NWUS extract served as the negative control. Microscopic examination revealed no notable differences in cell morphology between the NWUS and control groups (Fig. 3A and fig. S25A). Quantitative viability analysis confirmed that the relative cell viability in the NWUS group was statistically indistinguishable from the control, suggesting that NWUS is noncytotoxic (Fig. 3B and fig. S25B).

For in vivo evaluation, NWUS was implanted via a minimally invasive procedure around the right cervical vagus nerve in rats (Fig. 3C). Briefly, the electrode-exposed right side of the NWUS was passed beneath the isolated vagus nerve. The soft device was then wrapped tightly in a loop-by-loop manner, and the exposed SHPE on the left side was adhered to the previously wrapped portion of the device. The device remained stably positioned without displacement after 4 weeks, confirming its mechanical robustness and suitability for chronic implantation (Fig. 3D). Although localized hemorrhage was observed at the 4-week end point, this finding was attributed to the acute surgical dissection required to remove fibrous connective tissue for device visualization, rather than to chronic device-induced pathology. Furthermore, in vitro testing of NWUS wrapped around a 300-μm rod and incubated in 37°C PBS for 4 weeks showed no signs of swelling or delamination, further confirming the long-term structural integrity and stability of the device (fig. S26).

Postimplantation, vagus nerve tissues in direct contact with the device were harvested for immunofluorescence analysis. Key biomarkers were used to identify specific cell types and assess immune activation: Neuronal nuclei (NeuN) and S100 calcium-binding protein β (S-100β) were used to label neurons and Schwann cells, respectively, whereas tumor necrosis factor–α (TNF-α) was used as an indicator of immune response (Fig. 3E). No significant differences were observed in neuronal or Schwann cell populations between the NWUS and sham groups. Similarly, TNF-α expression remained at baseline levels, further supporting the absence of NWUS-induced neuroinflammation (Fig. 3F). Systemic biocompatibility was also assessed through histological analysis of major organs via hematoxylin and eosin (H&E) staining, which showed no pathological abnormalities throughout the implantation period (fig. S27). In addition, after 20 min of stimulation, the temperature at the stimulation site increased by only 1°C (Fig. 3G), indicating a low risk of thermal damage. Collectively, these findings demonstrate that NWUS has excellent biocompatibility and safety, supporting its potential for long-term implantation and reliable in vivo neuromodulation.

### NWUS-enabled neuromodulation for EAM treatment

To rigorously determine the optimal parameters for neuromodulation, we characterized the electrical output of the NWUS under physiological loading conditions. Therapeutic validation using a lipopolysaccharide (LPS)–induced acute inflammation model established a clear dose-response relationship between acoustic pressure and anti-inflammatory efficacy (fig. S28), identifying 150 kPa as the optimal pressure for cytokine suppression. When connected to the rat vagus nerve, NWUS generated a stimulation current exceeding 180 μA under an acoustic pressure of 150 kPa (fig. S29). This current intensity was verified to be sufficient to elicit robust compound action potentials (CAPs) in the vagus nerve, as evidenced in Fig. 2J. Furthermore, to validate the device’s capability to modulate specific neural targets in vivo, we recorded stimulation-evoked potentials under the optimized acoustic pressure of 150 kPa. The NWUS elicited robust CAPs in the vagus nerve (Fig. 2, J and K) and distinct compound muscle action potentials (CMAPs) in the sciatic nerve–gastrocnemius muscle circuit (Fig. 2L and fig. S30A). These findings confirm that the ultrasound-driven electrical output is sufficient to activate nerve fibers. Crucially, acute validation in an LPS-induced inflammation model (fig. S31) confirmed that this therapeutic efficacy stems from NWUS-mediated acoustic to electrical conversion rather than direct ultrasound modulation. Ultrasound exposure alone failed to significantly alter cholinergic anti-inflammatory pathway–related readouts, including splenic NE levels or proinflammatory cytokine levels, and did not produce measurable evoked CAPs (fig. S30B).

**Fig. 4.**
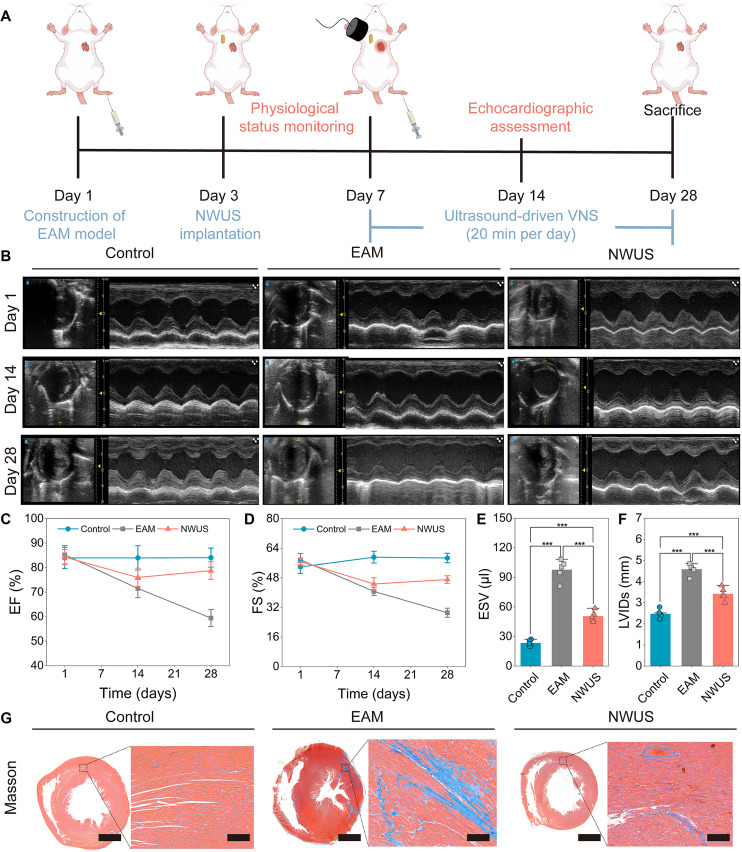
Chronic VNS via ultrasound-driven NWUS achieves effective EAM therapy. (**A**) Experimental timeline for EAM model induction and ultrasound-driven NWUS electrical stimulation treatment. (**B**) Echocardiographic imaging of rats in the control, EAM, and NWUS groups at days 1, 14, and 28 after immunization. (**C** and **D**) LV EF (C) and FS (D) at days 1, 14, and 28 postoperation (*n* = 5 independent animals). (**E** and **F**) ESV (E) and LVIDs (F) of hearts in the control, EAM, and NWUS groups at day 28 after modeling (*n* = 5 independent animals). (**G**) Masson’s staining of hearts in the control, EAM, and NWUS groups at day 28 after modeling, with blue indicating collagen deposition. Scale bars, 2 mm (left) and 100 μm (right). Data are presented as means ± SD in (C) to (F) and were analyzed by two-way ANOVA first and then by Tukey’s post hoc test. ***P ≤ 0.001.

Evaluation of the therapeutic efficacy of the wireless, ultrasound-driven NWUS system for chronic neuromodulation was then carried out using a rat model of EAM. EAM was induced on day 1 by subcutaneous immunization with porcine cardiac myosin (PCM) emulsified in Freund’s complete adjuvant (FCA). PCM primes autoreactive T cells against myocardial antigens, while FCA, containing inactivated *Mycobacterium tuberculosis*, activates innate immunity via Toll-like receptor signaling pathways. Following immunization, NWUS devices were surgically implanted around the right cervical vagus nerve on day 3. From days 7 to 28, animals received daily 20-min ultrasound-driven NWUS stimulation to wirelessly activate VNS. Photographs of the in vivo treatment setup are presented in fig. S32, showing the ultrasound transducer placed in direct contact with the skin overlying the implantation site to ensure effective acoustic coupling. EAM control animals underwent identical immunization and implantation procedures but did not receive ultrasound stimulation, while sham controls were administered FCA alone. Cardiac function was evaluated via echocardiography at designated time points, and histopathological examination was conducted at the study end point (Fig. 4A).

Body weight was continuously monitored as an indicator of systemic disease progression and therapeutic efficacy. As expected, PCM immunization induced progressive weight loss in EAM animals, with a significantly greater reduction compared to the sham group, reflecting disease severity. The NWUS group exhibited transient weight loss within the first 3 days postsurgery, likely due to reduced food intake associated with cervical incision recovery. Thereafter, NWUS-enabled VNS attenuated the ongoing weight loss, and by day 20, the body weight of the NWUS group had recovered to levels comparable to those of the control group (fig. S33). In addition, systemic inflammatory responses were evaluated by analyzing spleen–to–body weight ratio (Sw/Bw) and heart–to–body weight ratio (Hw/Bw) at the experimental end point. Representative spleen images showed marked splenomegaly in the EAM group compared to controls, which was partially alleviated in the NWUS-treated group (fig. S34A). Quantitative analysis revealed that absolute spleen weight (fig. S34B), Sw/Bw (fig. S34C), and Hw/Bw (fig. S34D) were significantly elevated in the EAM group relative to controls. NWUS treatment significantly reduced both Sw/Bw and Hw/Bw ratios, indicating attenuation of systemic and cardiac inflammation. These findings support the anti-inflammatory efficacy of NWUS-enabled VNS in the EAM model.

Serial M-mode echocardiography revealed progressive left ventricular (LV) dilation and diminished wall motion in the EAM group, indicative of substantial systolic dysfunction (Fig. 4B). In contrast, rats treated with NWUS exhibited preserved LV geometry and contractile function throughout the observation period. Quantitative analysis revealed that by day 28, EAM substantially compromised cardiac function, with LV ejection fraction (EF) and fractional shortening (FS) declining to 59.4 ± 3.5% and 29.8 ± 2.5%, respectively, compared to control values of 84.1 ± 3.9% and 58.7 ± 2.5%. NWUS-enabled VNS effectively preserved cardiac function, markedly mitigating the decline in EF and FS values maintained at 78.7 ± 3.6% and 47.1 ± 2.1%, respectively (Fig. 4, C and D). Further structural evaluation supported these findings. By day 28, pronounced chamber remodeling was observed in the EAM group, reflected by increases in LV internal diameters at end-systole (LVIDs: 4.6 ± 0.3 mm) and end-systolic volume (ESV: 97.6 ± 10.5 μl). NWUS-enabled VNS attenuated these pathological changes, partially restoring LVIDs to 3.4 ± 0.3 mm and ESV to 50.5 ± 7.5 μl, although values remained elevated relative to the control group (LVIDs: 2.5 ± 0.2 mm; ESV: 23.4 ± 3.0 μl) (Fig. 4, E and F). Thus, preservation of contractile indices (EF and FS) alongside suppression of chamber dilation (LVIDs and ESV) suggests that NWUS-enabled VNS effectively delays adverse cardiac remodeling and functional deterioration in EAM.

Complementing the echocardiographic findings, histological analyses provided structural evidence supporting the attenuation of myocardial injury. H&E staining revealed diffuse inflammatory infiltrates and extensive myocyte damage in the EAM group. These pathological changes were substantially reduced in rats receiving NWUS treatment (fig. S35). Consistently, Masson’s trichrome staining showed extensive collagen deposition in the myocardium of EAM rats, indicative of severe interstitial fibrosis. In contrast, the NWUS group exhibited markedly reduced fibrotic remodeling, with myocardial architecture approaching that of healthy controls (Fig. 4G). These structural improvements, characterized by reduced immune cell infiltration and diminished collagen deposition, are consistent with the observed enhancements in LV function. Together, these findings suggest that NWUS-enabled VNS mitigates cardiac dysfunction and pathological remodeling by reducing myocardial inflammation, limiting leukocyte infiltration, and attenuating fibrotic progression.

### Assessment of anti-inflammatory and antifibrotic effects

**Fig. 5.**
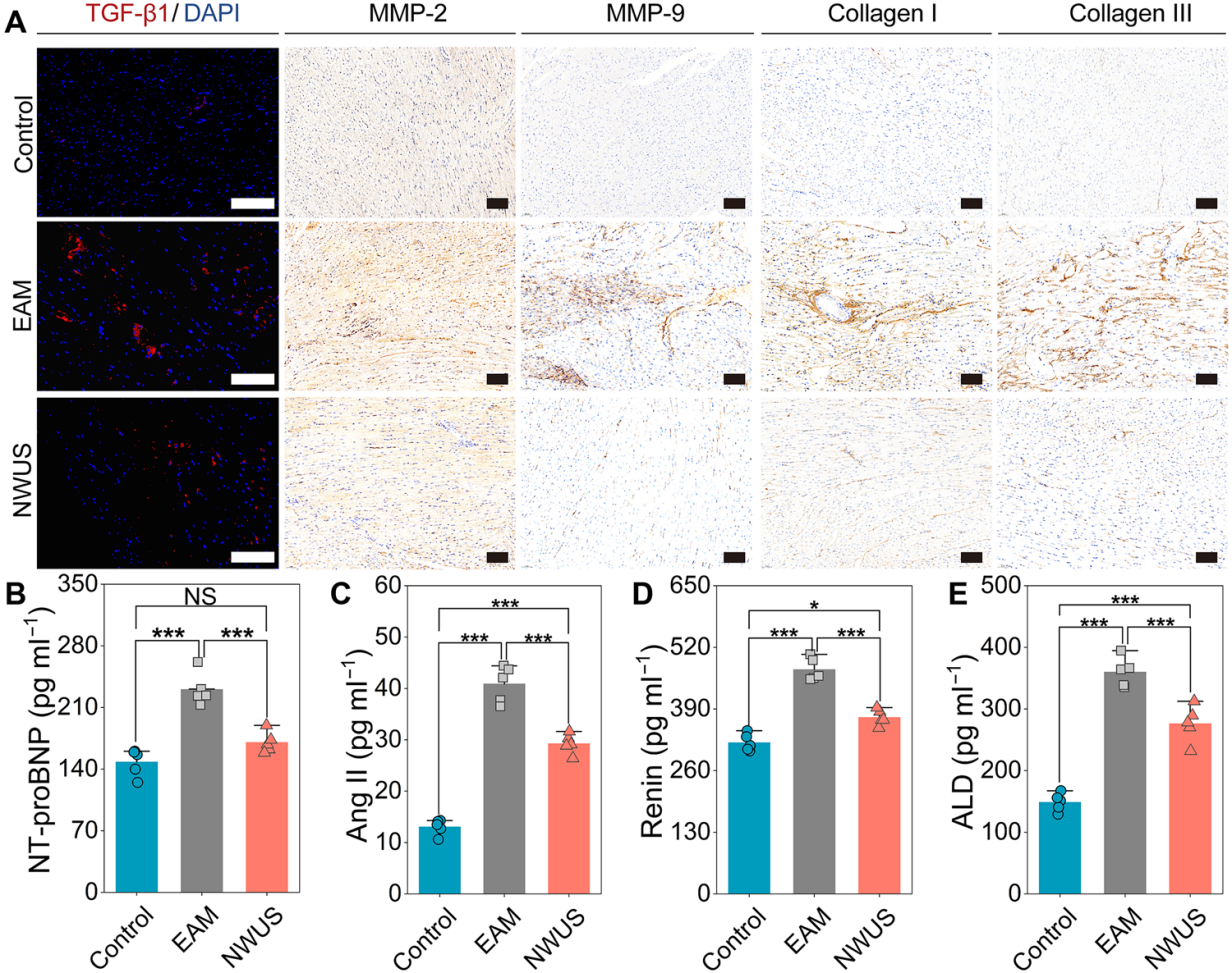
NWUS-enabled VNS mitigates myocardial fibrosis and cardiac remodeling. (**A**) Representative immunofluorescence images of TGF-β1 and representative immunohistochemistry images of MMP-2, MMP-9, collagen I, and collagen III in the control, EAM, and NWUS groups. Scale bars, 100 μm. (**B** to **E**) Quantification of serum levels of NT-proBNP (B), Ang II (C), renin (D), and ALD (E) (*n* = 5 independent animals). Data are presented as means ± SD in (B) to (E) and were analyzed by one-way ANOVA first and then by Tukey’s post hoc test. *P ≤ 0.05; **P ≤ 0.01; ***P ≤ 0.001.

Immunofluorescence and immunohistochemical analyses were further performed to evaluate the therapeutic potential of NWUS-enabled VNS in attenuating pathological cardiac remodeling. The EAM group displayed marked fibrotic alterations, characterized by elevated expression of the profibrotic cytokine transforming growth factor β1 (TGF-β1) and increased levels of matrix metalloproteinase-2 (MMP-2) and MMP-9, compared to the control group (Fig. 5A). These MMPs, typically involved in maintaining extracellular matrix (ECM) homeostasis, were markedly up-regulated under the chronic inflammatory conditions of EAM. This dysregulation reflects active ECM remodeling that drives myocardial fibrogenesis. Consistently, extensive interstitial deposition of collagen I and collagen III was observed, indicative of myocardial fibrosis and increased tissue stiffness. Quantitative analysis confirmed significantly larger immunopositive areas for all fibrotic markers in EAM rats (fig. S36, A to E). Following 21 days of NWUS-enabled VNS, expression levels of TGF-β1, MMP-2, MMP-9, collagen I, and collagen III were significantly down-regulated, partially restoring to levels observed in control animals. This coordinated down-regulation of fibrotic mediators suggests that NWUS may modulate multiple molecular pathways implicated in cardiac fibrotic remodeling. Specifically, reduced TGF-β1 expression may suppress fibroblast activation and subsequent collagen synthesis, while the partial restoration of MMP-2 and MMP-9 levels indicates a shift toward balanced ECM turnover (*41*, *42*). Collectively, these findings suggest that NWUS-enabled VNS can attenuate fibrotic progression and promote structural cardiac improvement in autoimmune myocarditis.

Serum biomarker profiling provides additional systemic evidence further substantiating the improvements in fibrosis and cardiac remodeling. In the EAM group, serum levels of N-terminal pro–B-type natriuretic peptide (NT-proBNP) (Fig. 5B) and cardiac troponin I (cTnI) (fig. S36F) were markedly elevated, reflecting increased cardiac stress and ongoing myocardial injury. NWUS-enabled VNS led to a significant reduction in both biomarkers, indicating improved cardiac function and diminished myocardial injury. Similarly, circulating levels of renin, angiotensin II (Ang II), and aldosterone (ALD), which are key effectors of the renin-angiotensin-aldosterone system (RAAS), were significantly elevated in EAM animals (Fig. 5, C to E). This elevation reflects the hyperactivation of the neurohormonal axis that promotes fibrosis and maladaptive cardiac remodeling (*43*, *44*). Specifically, NWUS-enabled VNS significantly reduced circulating levels of renin, Ang II, and ALD, suggesting a dampening effect on systemic RAAS activation.

### Assessment of local and systemic immune responses

**Fig. 6.**
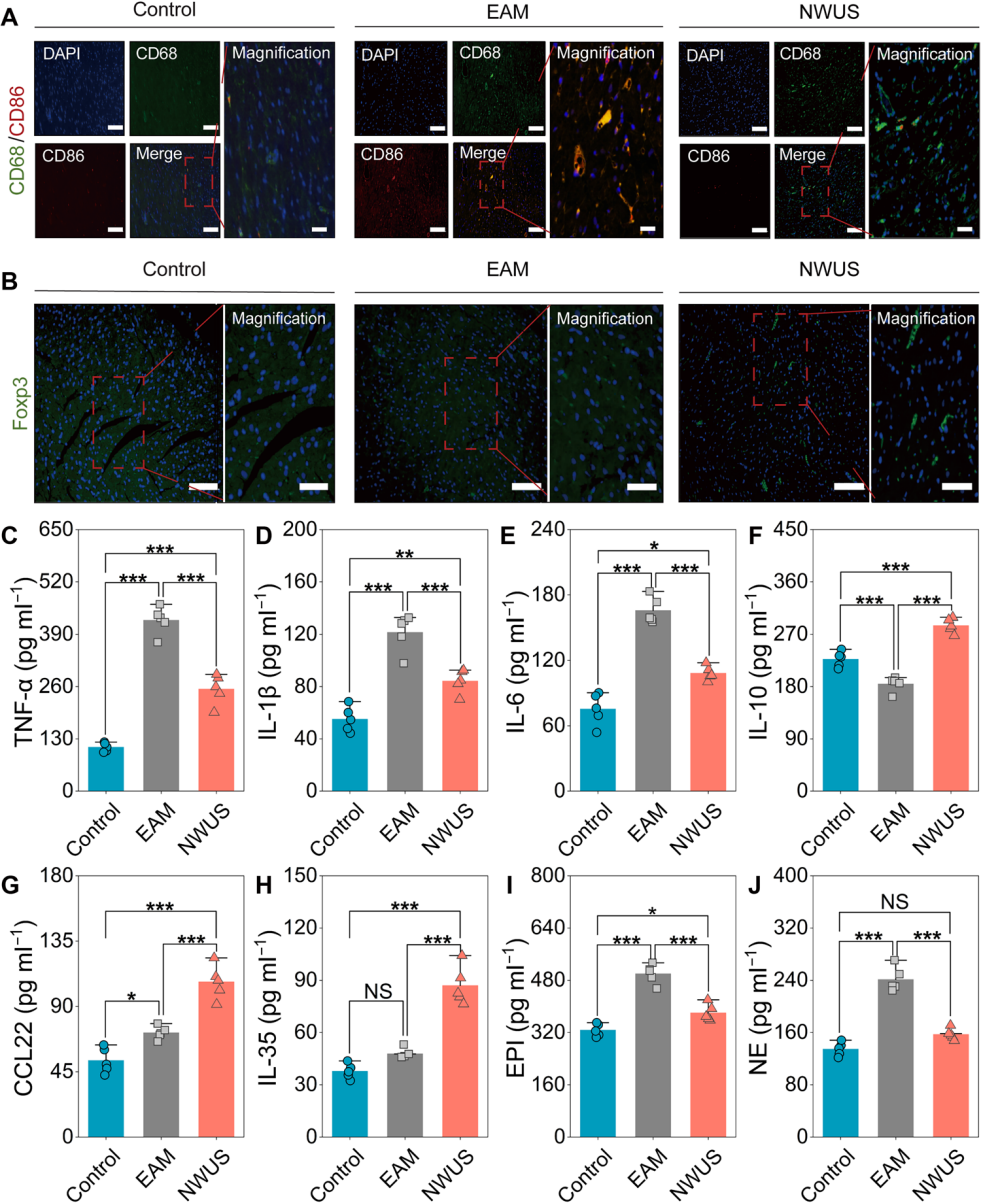
NWUS-enabled VNS modulates the immune response in EAM. (**A**) Representative immunofluorescence images of myocardial sections stained for CD68 (green) and CD86 (red), with DAPI nuclear counterstain (blue), in the control, EAM, and NWUS groups. Scale bars, 100 μm. Insets show high-magnification views of the merged channels. Scale bars, 20 μm. (**B**) Representative sections stained for Foxp3 (green) with DAPI (blue) in control, EAM, and NWUS groups. Scale bars, 100 μm. Insets show high-magnification views. Scale bars, 50 μm. (**C** to **J**) Quantitative analysis of TNF-α (C), IL-6 (D), IL-1β (E), IL-10 (F), CCL22 (G), IL-35 (H), NE (I), and EPI (J) (*n* = 5 independent animals). Data are presented as means ± SD in (C) to (J) and were analyzed by one-way ANOVA first and then by Tukey’s post hoc test. *P ≤ 0.05; **P ≤ 0.01; ***P ≤ 0.001.

Next, in-depth immunofluorescence analysis revealed that NWUS-enabled VNS profoundly modulated the immune cell landscape within the EAM myocardium (Fig. 6, A and B). In the EAM group, myocardial tissue was extensively infiltrated by CD68^+^ macrophages coexpressing CD86, a marker of the M1 proinflammatory phenotype (*45*). These M1 macrophages release nitric oxide and secrete a range of proinflammatory cytokines, exacerbating myocardial damage (*46*). By contrast, NWUS-treated cardiac tissue exhibited a marked reduction in CD68/CD86-positive macrophages, reflecting effective suppression of M1 macrophage polarization. In parallel, regulatory T cells (T_reg_ cells) expressing the transcription factor forkhead box P3 (Foxp3), which were sparse in untreated EAM hearts, were substantially enriched after NWUS-enabled VNS. These T_reg_ cells produce anti-inflammatory cytokines such as interleukin-10 (IL-10) and IL-35 and exert immunosuppressive effects to mitigate persistent inflammation. These reciprocal alterations, characterized by a reduction in proinflammatory macrophages and an increase in T_reg_ cell presence, indicate that NWUS-enabled VNS shifts the local immune milieu toward an anti-inflammatory and regulatory state. Mechanistically, the enhancement of vagal signaling may inhibit macrophage activation via α7 nicotinic acetylcholine receptors, while simultaneously promoting the expression of chemokines that facilitate T_reg_ cell recruitment (*47*–*49*). Together, these findings support that NWUS-enabled VNS modulates the myocardial immune microenvironment by suppressing innate inflammatory responses and facilitating recruitment of regulatory immune cells.

Systemically, NWUS-enabled VNS induced a robust anti-inflammatory response. Compared to the EAM group, circulating proinflammatory cytokines TNF-α, IL-6, and IL-1β were significantly decreased in the NWUS group (Fig. 6, C to E), suggesting effective suppression of systemic inflammation. In parallel, serum concentrations of IL-10, C-C motif chemokine 22 (CCL22), and IL-35 were substantially elevated posttreatment (Fig. 6, F to H), supporting enhanced T_reg_ cell–associated immunoregulation and regulatory-cell recruitment. NWUS treatment led to a significant reduction in circulating NE and epinephrine (EPI) (Fig. 6, I and J), reflecting inhibition of sympathetic nervous system activity (*50*, *51*). By attenuating sympathetic overactivation, it initiates a coordinated neuroimmune reprogramming that down-regulates proinflammatory pathways while enhancing regulatory immune responses. This dual modulation contributes to immune homeostasis in autoimmune myocarditis and underscores the therapeutic promise of bioelectronic medicine.

## DISCUSSION

This study presents the successful development and application of NWUS, an untethered, self-adhesive thin-film neurostimulator that enables wireless bioelectronic neuromodulation using externally programmable ultrasound pulses. NWUS integrates a high-efficiency triboelectric conversion mechanism with optimized impedance, a thin-film architecture with an excellent biocompatibility interface, and a self-adhesive SHPE encapsulation layer enabling nonstressed in vivo fixation of the device. These features enable the device to form a robust interface with microscale nerve trunks, such as the cervical vagus nerve in a rat model, which is ~250 μm in diameter. In a rat model of EAM, daily NWUS stimulation markedly improved LV function, reduced proinflammatory cytokine expression and macrophage infiltration within cardiac tissue, and promoted T_reg_ cell recruitment along with increased anti-inflammatory cytokine levels. In comparison with conventional neural stimulators, the NWUS system distinguishes itself by combining a wireless approach, untethered design, miniaturized architecture, and targeted neuromodulation. Owing to its unique architectural design, NWUS has the potential to be extended to other periorgan autonomic nerves, such as the splenic nerve, to address a broad spectrum of inflammatory disorders. The NWUS introduces an advanced paradigm in bioelectronic medicine, offering a versatile, precision tool for modulating previously inaccessible neural targets. Collectively, our results not only validate the therapeutic potential of NWUS in chronic autoimmune-driven cardiac disease, but also underscore its promise as a translational foundation for next-generation electroceutical bioelectronics.

## MATERIALS AND METHODS

### Construction of NWUS

NWUS was assembled from five functional layers via layer-by-layer stacking under cleanroom conditions. An SHPE film (9 mm by 7 mm, ~30-μm thick), serving as the outermost encapsulation layer, was prepared by reacting PTHF with isophorone diisocyanate and dibutyltin dilaurate in *N*,*N*-dimethylacetamide under nitrogen, end-capping with butanedione oxime, then casting, curing, and hot-pressing to yield the film. An Au/PEDOT:PSS foil (7 mm by 5 mm, ~10-μm thick), with PEDOT:PSS electrochemically deposited over a 7 mm–by–4 mm area by galvanostatic polymerization, then rinsed, dried, and immersed in ILs, was placed on the elastomer film 1.5 mm to the right of its center. A PU frame (7 mm by 4 mm, 5-μm thick), with a central 6 mm–by–3 mm opening, was aligned and adhered beneath the Au foil, exposing the PEDOT:PSS coating area. An Al foil (7 mm by 4 mm, ~15-μm thick) was placed beneath the PU frame, covering the exposed PEDOT:PSS area, ensuring that the stimulating electrode remained exposed. A PU film (7.5 mm by 5.5 mm, 5-μm thick), with two 0.6-mm square openings spaced 1.5 mm apart created via cutting, was adhered as the innermost layer, with the openings precisely aligned to expose the Au and Al foils, serving as the stimulation electrodes. To ensure measurement accuracy for the ultrathin device, the total thickness of the assembled NWUS was precisely quantified using a high-precision multifunction thickness tester (S16502, Frank-Pti, Germany).

### Measurement of the electrical performance

To characterize the electrical performance of ultrasound-driven NWUS, the device was wrapped around a 300-μm diameter silicone wire to simulate neural encirclement. It was then immersed in a water tank containing 1× PBS to mimic physiological conditions. The ultrasonic transducer was positioned directly in front of the NWUS, driven by a signal generator and power amplifier to induce triboelectric charge generation via acoustomechanical vibrations. Open circuit voltage was measured by connecting the positive electrode (Al contact) and negative electrode (Au contact) to a high-impedance oscilloscope (DHO1102, Rigol Technologies Co., Ltd., China). Short circuit current was assessed using a current amplifier (OE4102, Sine Scientific Instruments, China), with its output connected to the oscilloscope via Bayonet Neill-Concelman (BNC) connector, and NWUS’s stimulating electrodes linked to the current preamplifier’s inputs. To quantify the electrical output delivered to the target neural tissue, the NWUS was surgically connected to the rat cervical vagus nerve. The output voltage and current were recorded under varying acoustic intensities using an oscilloscope and a current amplifier, respectively, to simulate the actual impedance load of the nerve.

The acoustoelectric conversion efficiency (η) was determined based on the ratio of electrical output to acoustic input. The electrical output power density (*P*_out_) was calculated as the product of the open circuit voltage (*V*_oc_) and short circuit current (*I*_sc_), normalized by the effective contact area (18 mm^2^), according to the equation

$$P_{\text{out}}=\frac{V_{\text{oc}}\times I_{\text{sc}}}{A}$$

The acoustic input power density (*P*_in_) was derived from the measured acoustic intensity, which was calculated from the acoustic pressure (*p*) using the equation

$$P_{\text{in}}=\frac{p^{2}}{2\rho c}$$

where ρ and *c* represent the density (1000 kg m^−3^) and sound speed (1500 m s^−1^) of the water medium, respectively. The final conversion efficiency was obtained using η = (*P*_out_/*P*_in_) × 100%.

Power density was determined by connecting a variable load resistor (50 ohms to 10 megohms) in series, measuring voltage and current across various resistances, and calculating maximum power output relative to the 18-mm^2^ effective contact area defined by the overlap between the Al and PEDOT:PSS layers. To exclude electromagnetic interference during mechanism validation, the ultrasound transducer was wrapped in grounded aluminum foil, and all measurement leads were shielded and referenced to a common ground. The transferred charge density (σ) was calculated using the following equation

$$\sigma =\frac{\int I_{\text{sc}}\left(t\right)\mathrm{dt}}{A}$$

where *I*_sc_(*t*) represents the short circuit current over a single acoustic cycle, and *A* denotes the effective contact area between the functional triboelectric layers (the PEDOT:PSS coating and the Al foil).

### Construction of the EAM model

EAM was induced in Lewis rats using the standard cardiac myosin/FCA (*M. tuberculosis* H37Ra) protocol. All procedures were conducted in compliance with institutional guidelines and approved by the Institutional Animal Care and Use Committee of Tongji Medical College, Huazhong University of Science and Technology [(2025) IACUC no. 4796]. Male Lewis rats (8 to 10 weeks, 180 to 200 g; Beijing Vital River Laboratory Animal Technology Co., Ltd.) were housed under specific pathogen-free conditions and acclimatized for 3 days before immunization. PCM (Sigma-Aldrich, USA) was dissolved in PBS and emulsified 1:1 with FCA (Sigma-Aldrich, USA) containing heat-killed *M. tuberculosis* H37Ra (Gene-Optim, China). Each rat was subcutaneously injected at multiple sites with 0.4 ml of emulsion (myosin, 2.5 mg/ml) on day 1 and received a booster injection with the same preparation on day 7. Animals were monitored daily, and on day 28, hearts were collected for histological and molecular analyses to confirm myocarditis induction.

### Efficacy assessments after neuromodulation in the EAM model

Echocardiographic assessment was performed on days 1, 14, and 28 after immunization using a Vevo 3100 high-frequency imaging system (FUJIFILM VisualSonics, Canada). Rats were lightly anesthetized with 1.5 to 2.0% isoflurane in oxygen and placed on a temperature-controlled platform (37°C); heart rate was maintained between 350 and 450 bpm. Parasternal long- and short-axis views were acquired, and M-mode tracings were recorded. EF, FS, ESV, and LVIDs were measured and averaged over three to five consecutive cardiac cycles. All image acquisitions and analyses were performed blinded to treatment.

At the study end point, hearts were arrested in diastole (10% KCl), rinsed in cold PBS, and fixed in 4% paraformaldehyde (Servicebio, China). After paraffin embedding, H&E was used to assess inflammatory injury and necrosis, and Masson’s trichrome to quantify interstitial/perivascular fibrosis.

To evaluate cardiac remodeling, fibrosis, and local inflammation, immunohistochemistry was performed for MMP-2, MMP-9, collagen I, and collagen III to assess ECM turnover and deposition. Immunofluorescence staining was used to detect TGF-β1, a key fibrosis-related cytokine, as well as M1-like macrophages (CD68^+^CD86^+^) and T_reg_ cells (Foxp3^+^) to characterize the inflammatory microenvironment. Nuclei were counterstained with 4′,6-diamidino-2-phenylindole (DAPI). Major antibodies and reagents were purchased from Servicebio.

Peripheral blood was collected on day 28 for quantification of NT-proBNP and cTnI, as well as neurohumoral and inflammatory mediators Ang II, renin, ALD, TNF-α, IL-1β, IL-6, IL-10, CCL22, IL-35, EPI, and NE. All analytes were measured using enzyme-linked immunosorbent assay or chemiluminescent immunoassay kits (Servicebio, China) following the manufacturers’ protocols.

### Statistical analysis

All data are expressed as means ± SD. Statistical analyses were conducted using Origin 2023. Group differences were evaluated by one-way or two-way analysis of variance (ANOVA), followed by Tukey’s multiple comparisons test when appropriate. A two-sided *P* < 0.05 was considered statistically significant, with significance levels denoted as *P ≤ 0.05, **P ≤ 0.01, and ***P ≤ 0.001. NS indicates no significant difference. Each experiment was repeated at least three times independently.
