# Supplementary material for: Untethered thin-film neurostimulator wrapped around tiny nerve trunks for wireless neuromodulation
Source: Sci Adv. 2026 Jun 17;12(25):eaec9247. doi: 10.1126/sciadv.aec9247 (PMC13274598; doi:10.1126/sciadv.aec9247)
Supplement: Supplementary file 1 — Supplementary Text Figs. S1 to S36 Table S1 [file sciadv.aec9247_sm.pdf]

Science Advances
AAAS

Supplementary Materials for

Untethered thin-film neurostimulator wrapped around tiny nerve trunks for wireless neuromodulation

Renyuan Sun *et al.*

Corresponding author: Li Zhang, zli429@hust.edu.cn; Cunjiang Yu, cunjiang@illinois.edu; Zhiqiang Luo, zhiqiangluo@hust.edu.cn

Sci. Adv. **12**, eaec9247 (2026)
DOI: 10.1126/sciadv.aec9247

This PDF file includes:

Supplementary Text
Figs. S1 to S36
Table S1

Supplementary Text

Materials

Poly(tetrahydrofuran) (PTMEG) (Mw = 1000), isophorone diisocyanate (mixture of isomers, 99%), dibutyltin dilaurate (DBTDL, 95%), N,N-Dimethylacetamide (DMAc, ≥ 99.9%), dimethylglyoxime (98%) were purchased from Aladdin. 1× PBS (0.01 M, pH = 7.2~7.4) was purchased from Beijing Chreagen. The Cell Counting Kit-8 **(**CCK-8**)** and propidium iodide (PI, ≥ 94%) were purchased from Beyotime. Medium with high glucose (DMEM), penicillin-streptomycin solution (X100) were purchased from Hyclone. Fetal bovine serum was purchased from Umedium He Fei. Au foil (thickness, 0.4 μm) was purchased from Yong Bo. Al foil (thickness, 15 μm) was purchased from Biosharp. The DI water was collected using a Milli-Q water purification system (Millipore). All reagents were used as received, without purification. 3,4-ethylenedioxythiophene (EDOT, 97%) and poly(sodium 4-styrenesulfonate) (PSS, Mw = 70,000) were purchased from Sinopharm Chemical Reagent. Paraformaldehyde, sodium pentobarbital, and lipopolysaccharide (LPS) were purchased from Servicebio and Biosharp, respectively. Isoflurane was obtained from RWD Life Science. The inactivated lyophilized *Mycobacterium tuberculosis* H37Ra strain was purchased from Gene-Optim Biotechnology. Porcine cardiac myosin (PCM) and Freund's complete adjuvant (FCA) were obtained from Sigma-Aldrich.

Preparation of the Au/PEDOT:PSS foil

A three-electrode electrochemical setup was employed to fabricate Au/PEDOT electrodes using a CHI660E workstation (Shanghai Chenhua Instrument, China). An Au foil (0.4 μm thick, 10 × 10 mm) served as the working electrode and was attached to a PU film for mechanical support. A Pt plate (10 × 10 mm) served as the counter electrode, and an Ag/AgCl electrode served as the reference electrode. PEDOT was electrochemically deposited on Au foil via galvanostatic polymerization. A solution was prepared by dissolving 0.125 g (5 mg/mL) poly(sodium 4-styrenesulfonate) (PSS, Mw = 70,000) in 25 mL deionized water with continuous magnetic stirring until fully dissolved, followed by the addition of 25.7 μL (10 mM) 3,4-ethylenedioxythiophene (EDOT, 97%). The solution was maintained at room temperature with constant stirring. PEDOT:PSS coating was formed at a current of 40 μA for 900 s to ensure uniform deposition. After deposition, the Au/PEDOT:PSS foil was rinsed with deionized water and dried under high-purity nitrogen. To enhance electrical performance, the foil was immersed in the IL solution, prepared by dissolving 10 mL of 1-butyl-3-methylimidazolium tetrafluoroborate in a mixture of 10 mL deionized water and 10 mL Dimethyl sulfoxide (DMSO). The foil was then rinsed with deionized water and dried with high-purity nitrogen for subsequent use.

Characterization of the Au/PEDOT:PSS foil

SEM images of the Au/PEDOT:PSS foil were obtained using a field-emission scanning electron microscope (SEM, Nova NanoSEM 450, FEI, USA) after lyophilization using liquid nitrogen. Topography images of the Au/PEDOT:PSS foil were taken using an atomic force microscope (AFM, SPM9700, Shimadzu, Japan), and the data were analyzed using NanoScope Analysis. Quantitative analysis of elemental composition on the Au/PEDOT:PSS foil's surface was performed using X-ray photoelectron spectroscopy (XPS, K-Alpha, Thermo Fisher Scientific, USA). Long-term stability was assessed by immersing the foil in 0.1 M PBS at 37 °C for 4

weeks. EIS measurements were conducted by CHI660E workstation (Shanghai Chenhua Instrument, China) in the frequency range of 1 Hz to 1 MHz with an AC amplitude of 10 mV to monitor changes in charge-transfer resistance and phase angle.

Synthesis of SHPE

To synthesize the self-healing polyurethane elastomer (SHPE), 29 g of polytetrahydrofuran diol (PTMEG, MW = 1000) was stirred at 100 °C in a three-neck flask, vacuum-dried for 1 h, then cooled to 70 °C under nitrogen. A solution of 13.54 g isophorone diisocyanate (IPDI) and 0.10 g dibutyltin dilaurate (DBTDL) in 10 mL N,N-dimethylacetamide (DMAc) was added dropwise, and the mixture was maintained at 70 °C for 2 h. After cooling to room temperature, 3.366 g butanedione oxime was introduced and the reaction continued at 40 °C for 12 h. Finally, the product was dissolved in 60 mL DMAc to yield a 30 wt % solid content solution. For thin film preparation, the solution was cast into a polytetrafluoroethylene mold, heated at 130 °C for 12 h, and cooled to room temperature. This cycle was repeated five times to obtain a bubble-free film, which was hot-pressed at 70 °C for 60 min to ~30 μm thickness and sealed for storage.

Characterization of SHPE

The chemical structure of SHPE was studied using nuclear magnetic resonance spectroscopy (NMR, Avance III HD, Bruker, Switzerland) and Fourier transform infrared spectroscopy (FT-IR, Nicolet iS50R, USA).

A 30 wt% DMAc solution of SHPE was cast into a square Teflon mold, heated from 40 to 130 °C over 24 h, and vacuum-dried at 60 °C for 12 h to yield bubble-free samples. Mechanical properties were assessed using a universal testing machine (CTM8000, Xie Qiang Instrument Manufacturing, China). Tensile tests on dumbbell-shaped samples (30 mm × 5 mm × 2 mm) at 50 mm·min^{-1} determined the elastic modulus from the stress-strain curve's linear region.

The shear modulus was tested using a rheometer (MCR102, Anton Paar, Austria) set to operate at 37 °C. Angular frequency sweep measurements were conducted over a range from 0.1 to 10 rad s^{-1} with a 0.5% amplitude. The shear modulus was calculated using Equation (1):

$$E = 2\sqrt{G'^2 + G''^2} \cdot (1 + \nu) \tag{1}$$

Where E represents the shear modulus, G' and G'' represent the storage and loss moduli at 1 Hz, respectively, and ν, representing the Poisson's ratio, was assumed to be 0.5. Tensile tests were performed at a stretching rate of 1 mm·min^{-1}.

Self-healing was evaluated via scratch recovery, using an 18G needle to create intersecting scratches on films, tracked by optical microscopy. Mechanical restoration involved pressing two specimens (15 mm × 5 mm × 2 mm) together at room temperature, followed by lap shear tests at 20 mm·min^{-1}. Interfacial adhesion between the SHPE-SHPE and SHPE-PU layers was evaluated using the peeling test at 20 mm min^{-1}. Interfacial toughness was twice the peeling force divided by overlap width. Cyclic tensile tests were conducted for 1,000 cycles at a constant strain amplitude to evaluate mechanical durability and fatigue resistance.

Simulation and measurement of acoustic field

The acoustic-structural interaction was modeled in COMSOL Multiphysics by first selecting a three-dimensional geometry and enabling the "Solid Mechanics" and "Pressure Acoustics, Frequency Domain" interfaces. A 40 mm-diameter, 20 mm-tall cylinder representing muscle tissue was created, above which a 30 mm-diameter thin-film transducer was positioned flush with the cylinder's top surface. A 10 mm-long nerve surrogate (diameter 0.2 mm) was placed coaxially 5 mm beneath the transducer, and a ring-shaped device represented as NWUS (outer diameter 0.4 mm, inner diameter 0.2 mm, axial length 5 mm) was concentrically located about the mid-region of the nerve cylinder; each domain was assigned appropriate acoustic and density properties. The muscle base was constrained with a fixed-constraint boundary, while all remaining solid boundaries were left free. The fluid domain within the muscle cylinder was treated as an equivalent soft-tissue continuum for acoustic propagation, with the transducer–tissue interface driven by a spatially uniform normal acceleration corresponding to 150 kPa of acoustic pressure. The "Acoustic-Structure Boundary" multiphysics coupling was enabled to capture bidirectional interaction between the pressure field and structural deformation. A frequency-domain study was configured to solve for the prescribed excitation at the 500 kHz frequency.

The ultrasonic excitation apparatus comprised a function generator (AFG3021C, Tektronix, USA), a power amplifier (ATA-2021H, Aigtek, China), and an ultrasound transducer (DYW-500, Anbuleila Ultrasound, China). The function generator configured the frequency, pulse period, pulse interval, and amplitude of the pulsed ultrasound. The programmed pulse signal, amplified by the power amplifier, delivered enhanced output power to the transducer, thereby enabling ultrasonic wave generation. Ultrasonic measurement employed a hydrophone, a water tank, and a control system. The transducer's acoustic field was assessed by positioning a calibrated hydrophone within a water-filled tank to map the ultrasound field distribution emitted from the transducer surface, thereby yielding quantitative values of sound pressure and intensity. For testing, the transducer was mounted beneath the tank and received a predefined pulse voltage waveform from the function generator. This signal, amplified by the power amplifier, drove the ultrasonic probe affixed in the tank. The hydrophone, placed directly ahead and aligned with the transducer's center, captured the acoustic field distribution, thereby determining the sound pressure and intensity of the emitted ultrasound.

Cytocompatibility assay

The cytocompatibility of NWUS was evaluated using CCK-8 and live-dead staining assays with L929 and PC12 cell lines. NWUS was immersed in Dulbecco's modified Eagle medium (DMEM) at 37 °C for 24 h to obtain the extract (100 mg NWUS in 1 mL DMEM). L929 and PC12 cells were seeded in 24-well plates at densities of 3000, 2000, and 1500 cells/well, respectively, and incubated at 37 °C with 5% CO_2 for 1, 3, and 5 days. Cell viability was quantified via CCK-8 assay. Additionally, cells were seeded in 6-well plates at 10,000, 6000, and 6000 cells/well for L929 and PC12, respectively, and cultured under identical conditions for 1, 3, and 5 days. Cell morphology was examined using live-dead staining.

Physiological safety of NWUS-Enabled VNS

Body temperature and heart rate of rats were monitored at baseline and at 10- and 20-min during stimulation. Core body temperature was assessed using an infrared thermal imaging camera (E50, FLIR, USA).

Efficacy assessments of NWUS-enabled nerve stimulation

Electrophysiological signals were collected using a biological signal acquisition system (BL-420N, Chengdu Tai Meng Software Co., Ltd.). To mimic physiological conditions, NWUS was wrapped around a 200 μm diameter cylinder and submerged in a water tank. An ultrasonic transducer, driven by a signal generator and amplifier, was placed 5 mm directly in front of NWUS. The vagus nerve was exposed, connected to NWUS's stimulating electrodes via copper wires, and stimulated while nerve action potentials were recorded before and after stimulation. In a separate procedure, the sciatic nerve was similarly exposed and interfaced with NWUS's electrodes via copper wires. Needle electrodes, inserted into the gastrocnemius muscle, were attached to the system's signal input. Ultrasonic signals from the transducer were transformed by NWUS into electrical stimuli applied to the sciatic nerve, and muscle action potential changes before and after stimulation were recorded. The gastrocnemius tendon was then isolated, sutured to the system's tension sensor, and stimulated, with muscle tension variations noted.

Lipopolysaccharide (LPS) was dissolved in saline at a concentration of 2 mg/mL and sterilized via filtration through a 0.22 μm syringe filter. Systemic inflammatory response syndrome (SIRS) was induced by administering the sterile LPS solution through the femoral vein at a dosage of 10 mg/kg.

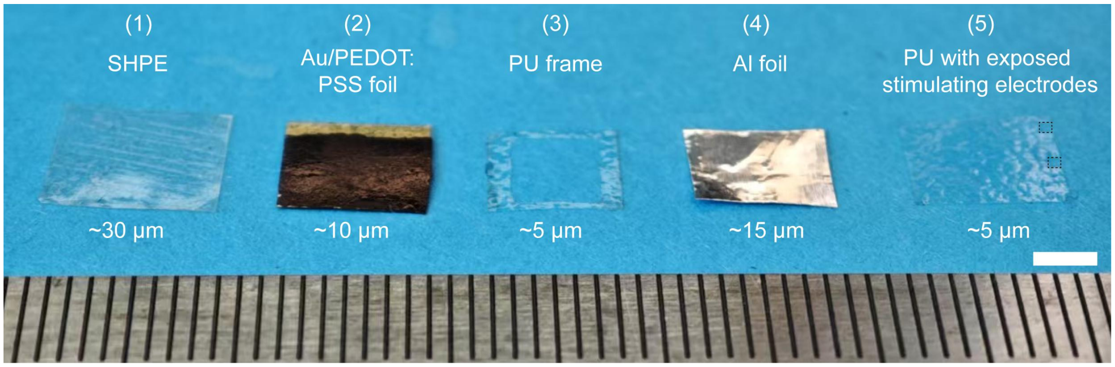

Fig. S1. Photograph of the five-layer functional structure of NWUS. NWUS is constructed from a five-layer architecture comprising: (1) a ~30 µm self-healing polyurethane elastomer (SHPE) outer layer (9 mm × 7 mm) for conformal wrapping on nerve; (2) an Au/PEDOT:PSS composite electrode (7 mm × 5 mm) formed via electrochemical deposition, serving as one triboelectric material; (3) a polyurethane (PU) spacer (7 mm × 4 mm) with a central 6 mm × 3 mm opening enabling contact-separation motion; (4) an aluminum (Al) foil layer (7 mm × 4 mm) as the counter triboelectric surface; and (5) an inner PU adhesive film (7.5 mm × 5.5 mm) that seals the device. Exposed Au and Al electrodes interface directly with the nerve to deliver stimulation. Scale bar, 3 mm.

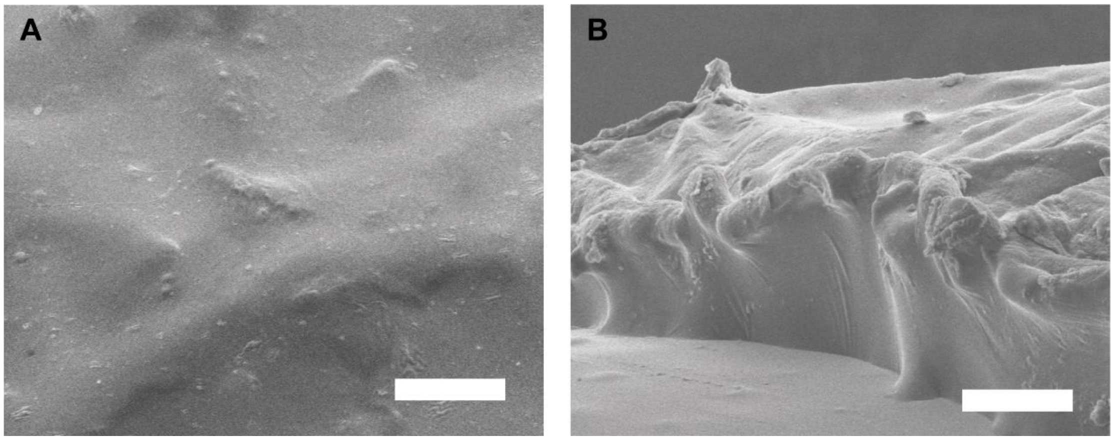

Fig. S2. SEM image of the Au/PEDOT:PSS foil. (A) Surface SEM image of the Au/PEDOT:PSS foil. **(B)** Cross-sectional SEM image of the Au/PEDOT:PSS foil. Scale bar, 10 μm.

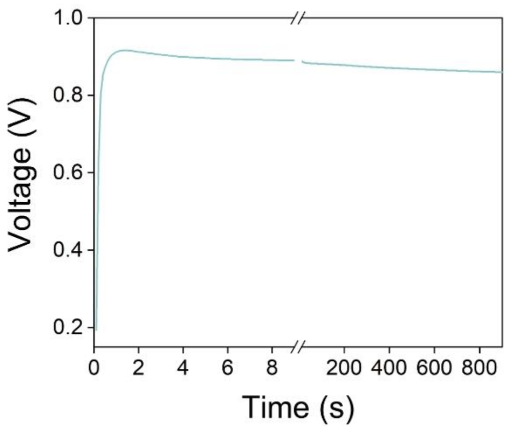

Fig. S3. Electrodeposition of PEDOT:PSS on Au foil. Chronoamperometric curve of PEDOT:PSS electrodeposited at constant current for 900 seconds.

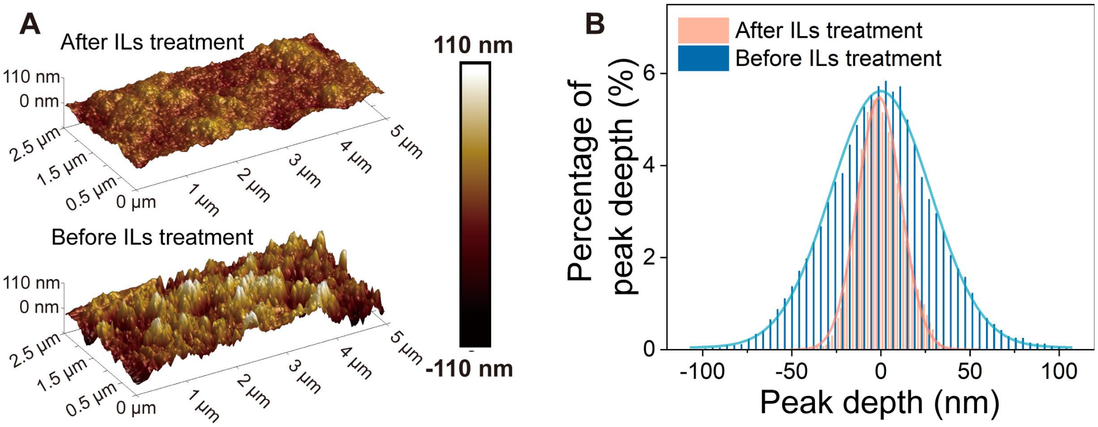

Fig. S4. Surface morphology characterization of Au/PEDOT:PSS foil before and after ILs treatment. **(A)** Representative AFM images showing topographical changes. **(B)** Quantitative analysis of surface peak height distribution.

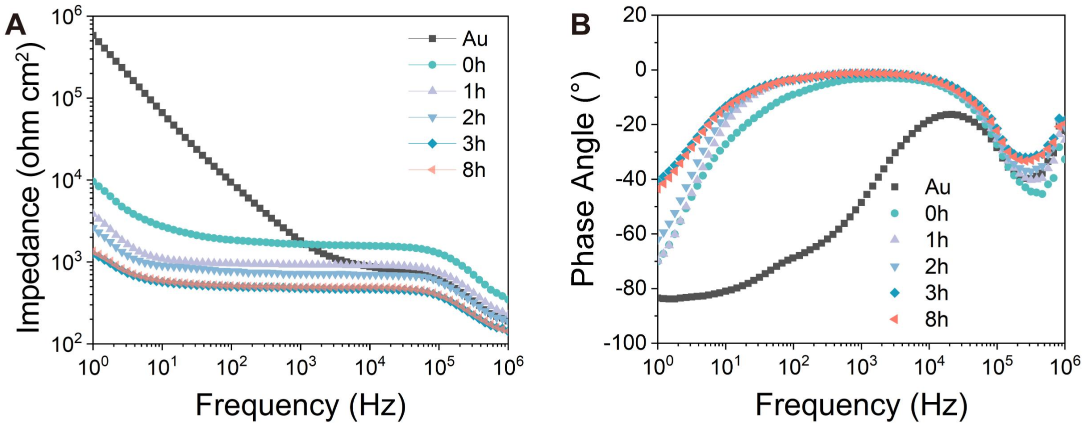

Fig. S5. Electrochemical impedance spectroscopy (EIS) of the Au/PEDOT:PSS foil after varying durations of ILs treatment. (A) A progressive decrease in charge transfer resistance with longer treatment times. **(B)** An increase in phase angle in the high-frequency region with longer treatment times.

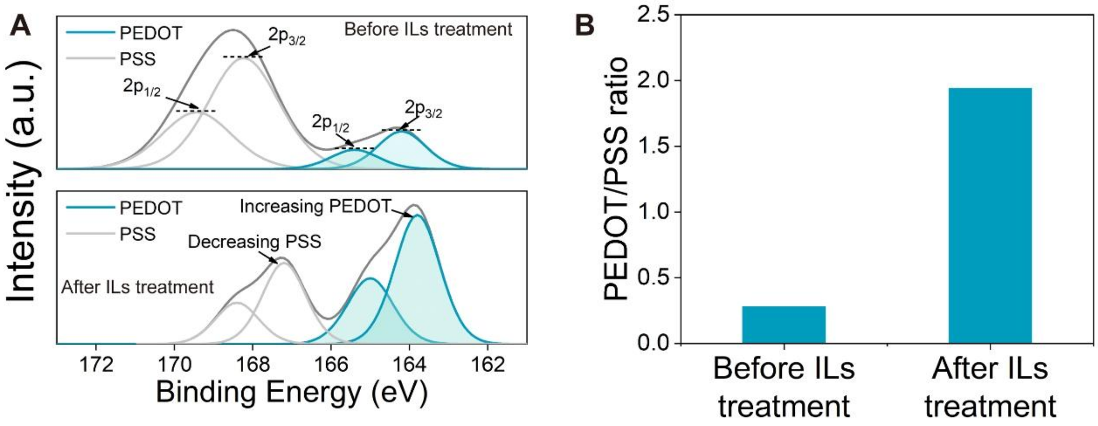

Fig. S6. XPS spectra of the S 2p orbital in the Au/PEDOT:PSS foil. (A) The Au/PEDOT:PSS foil exhibited sulfur S 2p characteristic peaks of sulfur atoms in PEDOT (161-166 eV) and PSS (166-170 eV). **(B)** Quantitative PEDOT/PSS ratio analysis in the Au/PEDOT:PSS foil before and after ILs treatment.

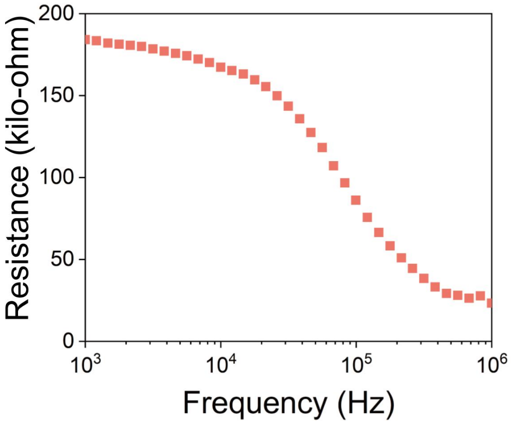

Fig. S7. Resistance measurement of a 1 mm long rat vagus nerve across frequencies ranging from 1 kHz to 1 MHz by electrochemical workstation. It shows a decrease from approximately 180 kilo-ohms to 18 kilo-ohms.

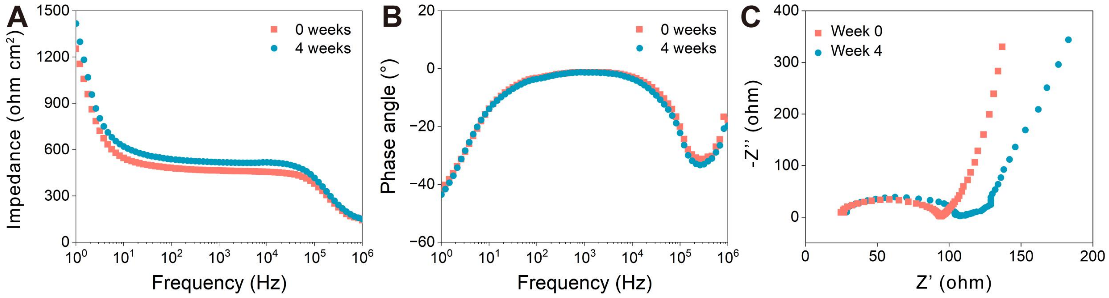

Fig. S8. Electrochemical impedance spectroscopy (EIS) and Nyquist plots of the Au/PEDOT:PSS foil before and after 4 weeks soaking in PBS. (A) Impedance magnitude changes before and after immersion. **(B)** Phase angle shifts before and after immersion. **(C)** Nyquist plots before and after immersion.

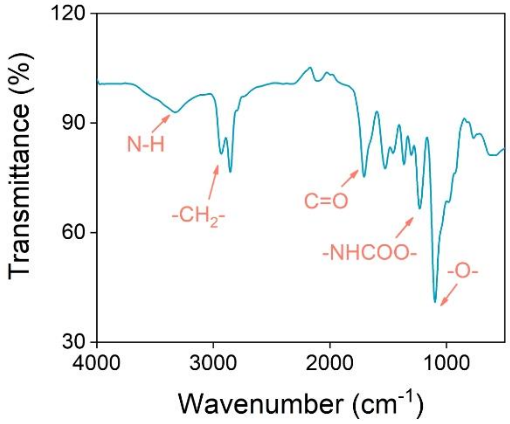

Fig. S9. FT-IR spectrum of SHPE. It shows characteristic peaks assigned to key functional groups of SHPE.

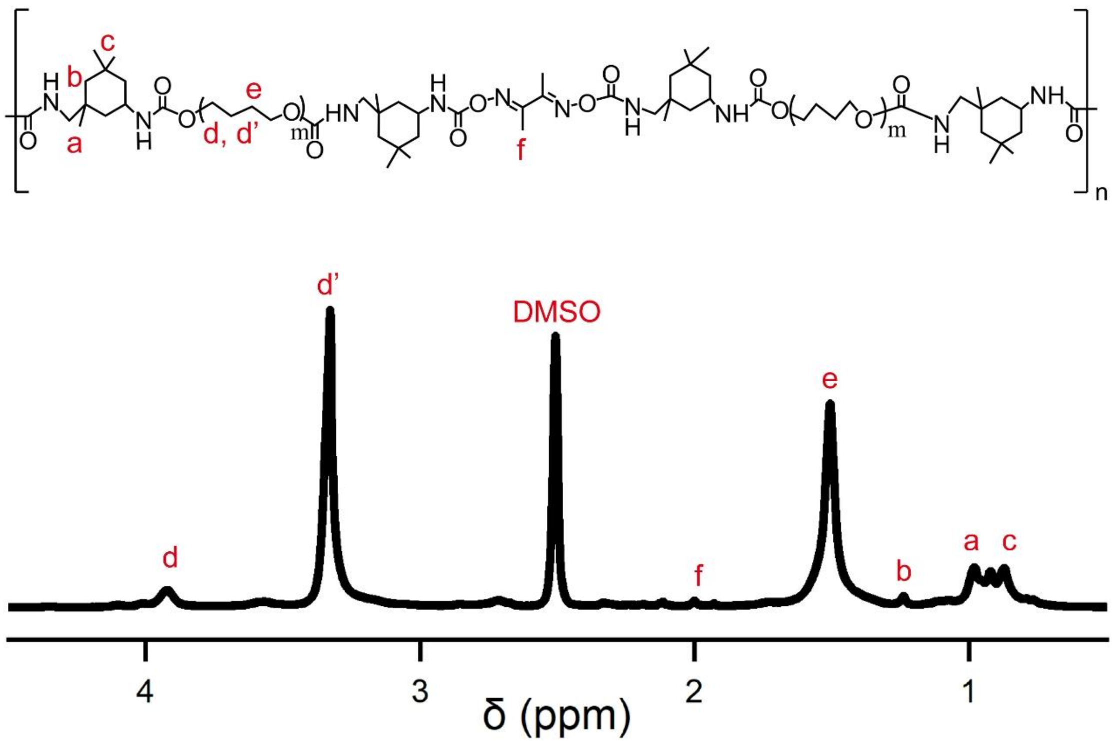

Fig. S10. NMR spectrum of SHPE. The 1H NMR of the developed SHPE confirms its successful synthesis and supports the functional group assignments identified by FT-IR analysis.

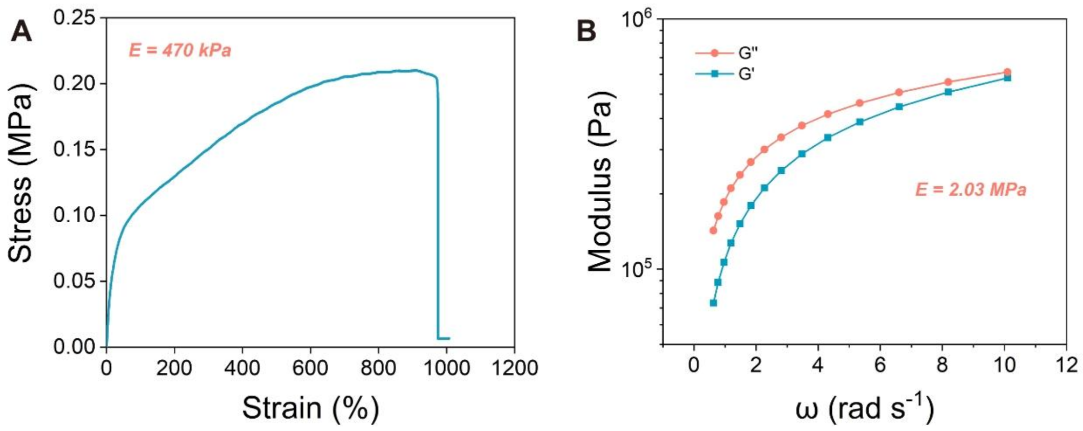

Fig. S11. Mechanical properties of SHPE. (A) Representative tensile stress-strain curve of the SHPE. **(B)** The rheological variation of G′ and G″ for SHPE reveals a shear modulus of 2.03 MPa.

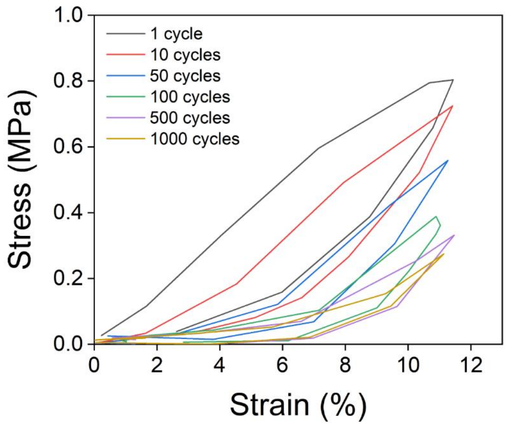

Fig. S12. Cyclic tensile test of SHPE repeated with 1,000 cycles. The stress-strain curves show stabilization after initial cycles and no fracture, indicating mechanical durability suitable for chronic implantation.

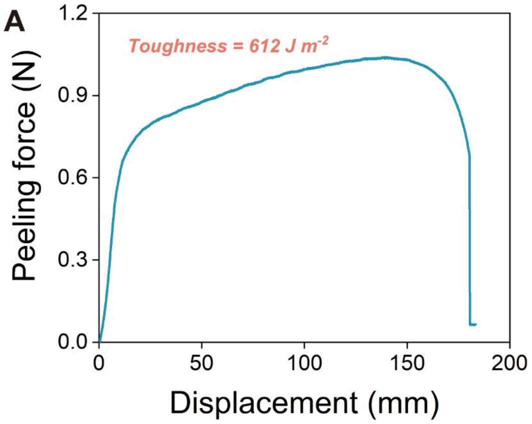

Fig. S13. Peeling test results of the SHPE film and the SHPE-PU interface. (A) Peeling test results of the SHPE-SHPE interface showing a post-healing interfacial adhesion energy of 612 J m^{-2} after 2 minutes of self-healing. **(B)** Peeling test results of the SHPE-PU interface, showing an adhesion energy of 427 J m^{-2}, confirming robust encapsulation stability.

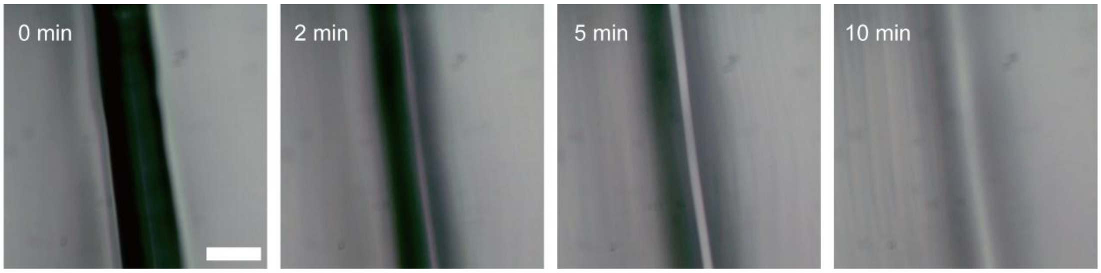

Fig. S14. Optical images of the damaged and healed SHPE film. It shows the disappearance of the groove after 10 min healing at room temperature. Scale bar, 100 μm.

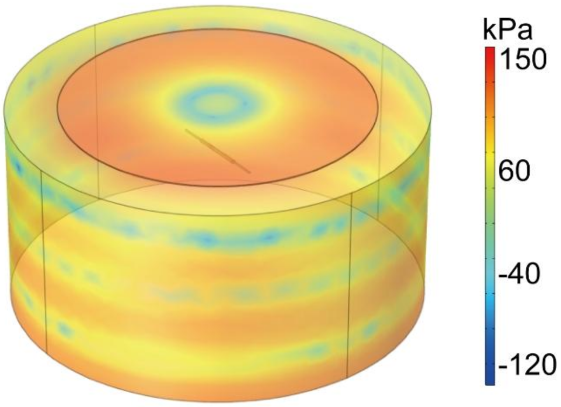

Fig. S15. COMSOL simulation of acoustic pressure distribution under ultrasound excitation with 150 kPa acoustic pressure.

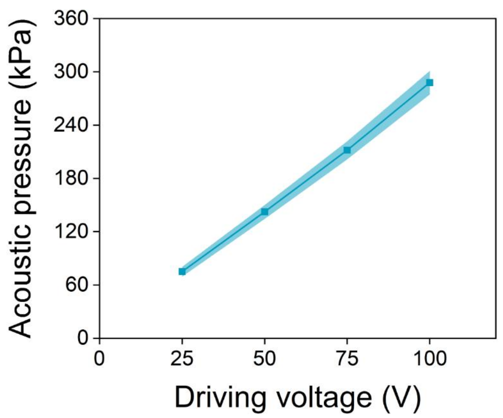

Fig. S16. Acoustic pressure (measured in water) generated from ultrasonic transducer driven by various voltages. Voltages ranging from 25 V to 100 V produced acoustic pressure ranging from 75 to 281 kPa.

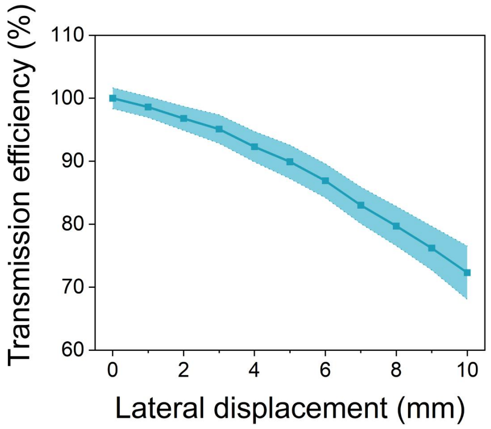

Fig. S17. Effect of lateral displacement of the hydrophone on measured acoustic pressure. It shows a reduction to 72.4% of the peak pressure at a 10 mm off-axis position.

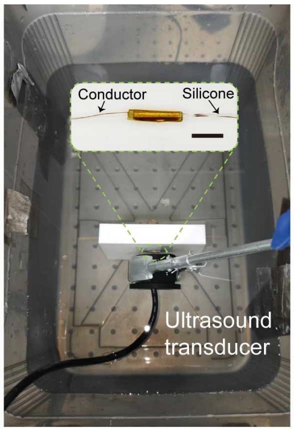

Fig. S18. Photograph of the setup for NWUS electrical performance testing. NWUS was wrapped around a silicone wire 300 μm in diameter and immersed in water, with the ultrasonic transducer positioned 5 mm away. Scale bar, 5 mm.

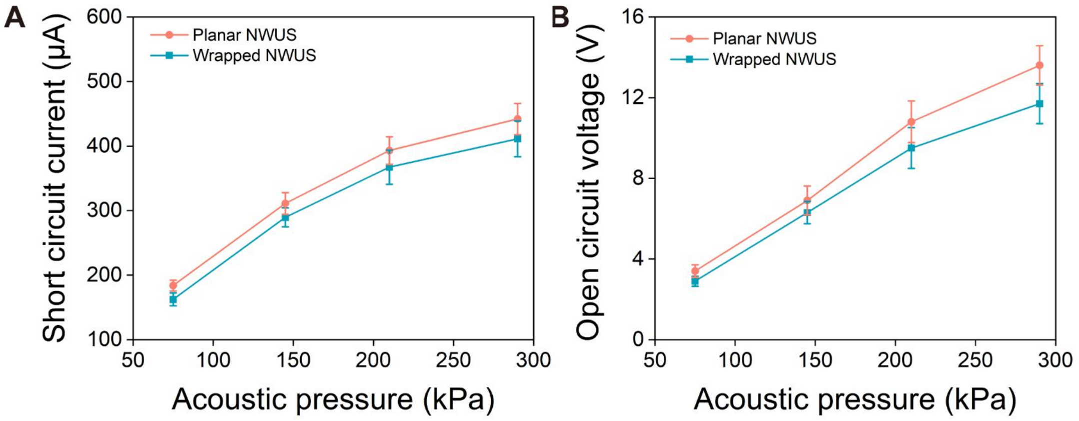

Fig. S19. Output performance testing of NWUS in different configurations. (A) Short circuit current of planar and wrapped NWUS under varying acoustic intensities. **(B)** Open circuit voltage of planar and wrapped NWUS under varying acoustic intensities.

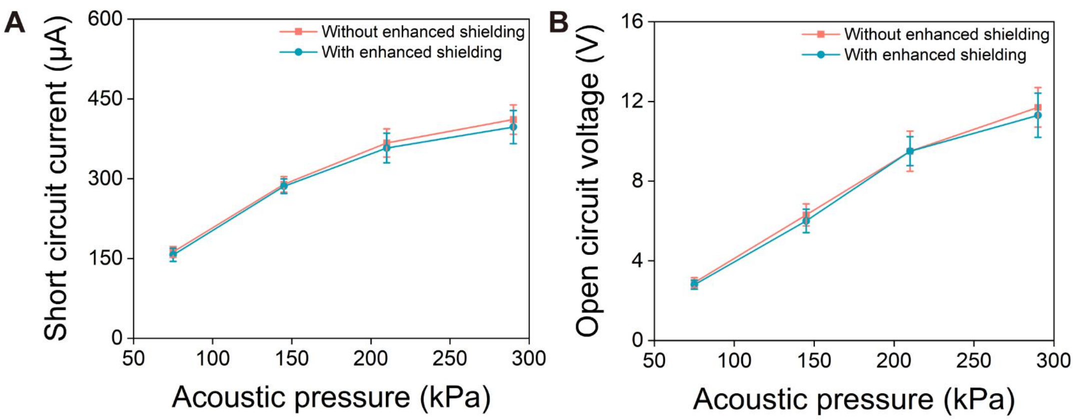

Fig. S20. Output performance of NWUS with and without enhanced shielding. (A) Short circuit current of NWUS with and without enhanced shielding. **(B)** Open circuit voltage of NWUS with and without enhanced shielding.

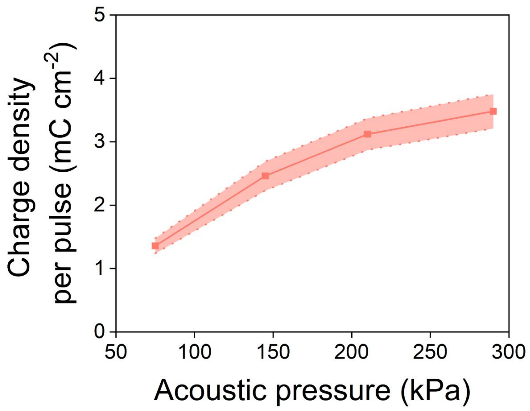

Fig. S21. Relationship between transferred charge density and acoustic pressure. The charge density exhibits a linear increase with acoustic pressure up to 210 kPa, followed by a plateau due to surface charge saturation.

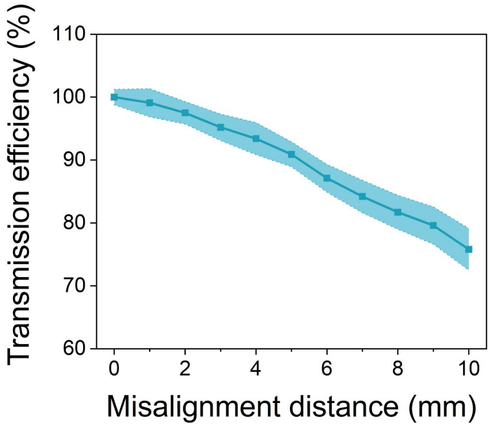

Fig. S22. Transmission efficiency under NWUS misalignment. Normalized power transfer efficiency measured at lateral offsets of 0-10 mm.

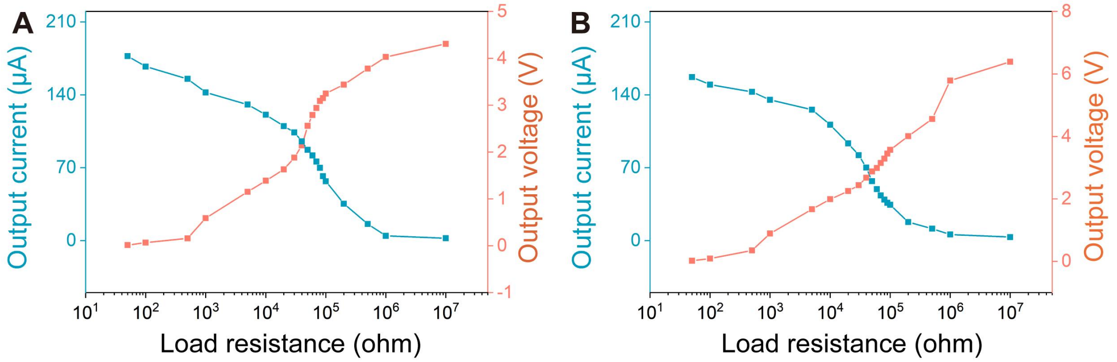

Fig. S23. Output voltage and current measured across load resistances ranging from 50 ohms to 10 mega-ohms. (A) Output voltage and current of NWUS before ILs treatment under different load resistances. **(B)** Output voltage and current of Au-Al TENG under different load resistances.

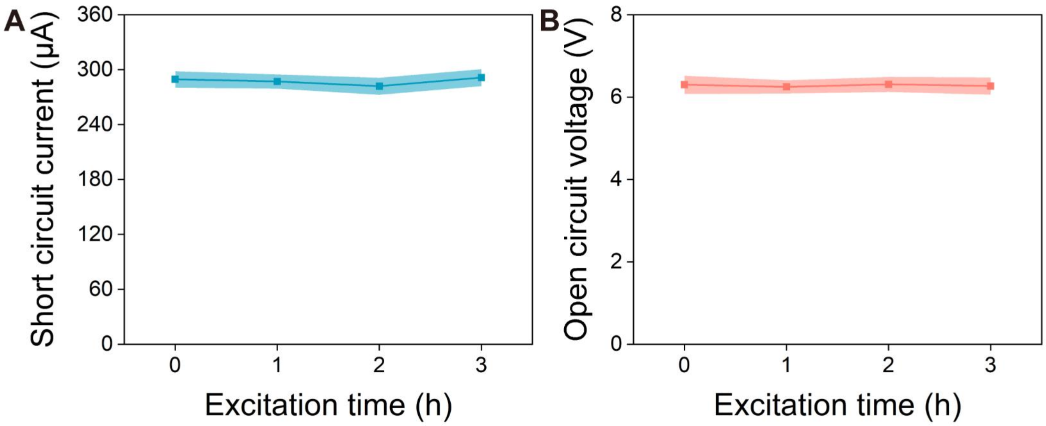

Fig. S24. Output performance stability under continuous ultrasound pulsing. (A) Output current and **(B)** output voltage measurements recorded over 3 hours of continuous excitation (2 ms pulse width, 1 s interval), showing no notable performance degradation.

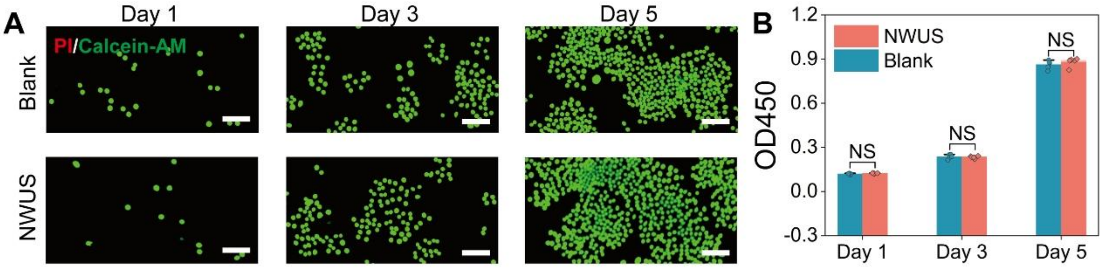

Fig. S25. ***In vitro*** **cytotoxicity assay of NWUS. (A)** Live-dead staining of L929 cells with the NWUS extracts. Scale bar, 100 μm. **(B)** Cell viability of L929 cells with the NWUS extracts (n = 5). Data are presented as the mean ± SD in **(B)** and were analyzed by one-way ANOVA first, and then by the Tukey's post hoc test. NS, not significant.

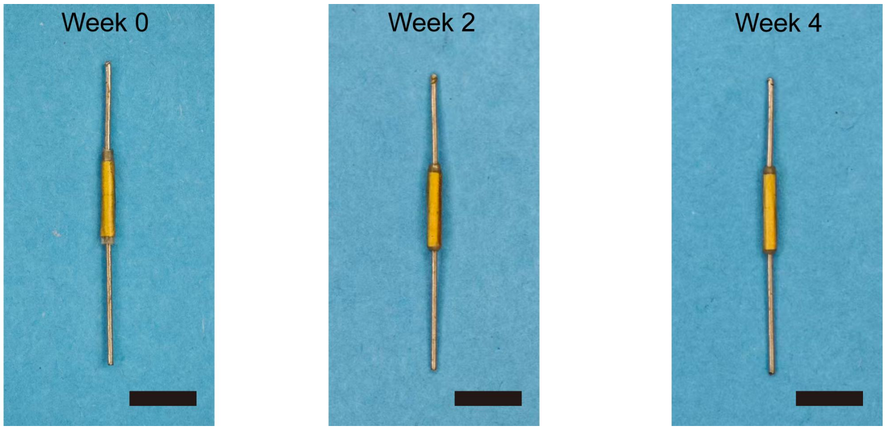

Fig. S26. *In vitro* structural stability of NWUS. Photographs of the NWUS conformally wrapped around a metal rod (~300 μm in diameter) after 4 weeks of immersion in PBS at 37 °C, showing maintained structural integrity with no signs of delamination or swelling. Scale bar, 5 mm.

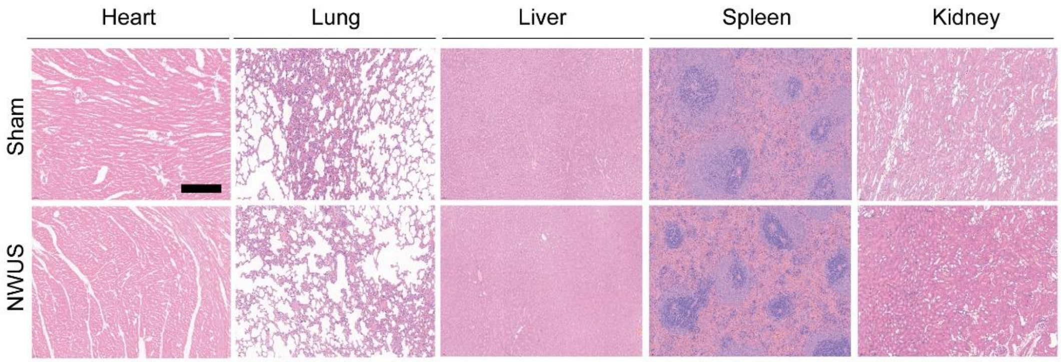

Fig. S27. Histological analysis of major organs (n = 5 independent animals). H&E staining of the heart, liver, spleen, lung, and kidney tissues in the VNS and Sham group. Scale bar, 100 μm.

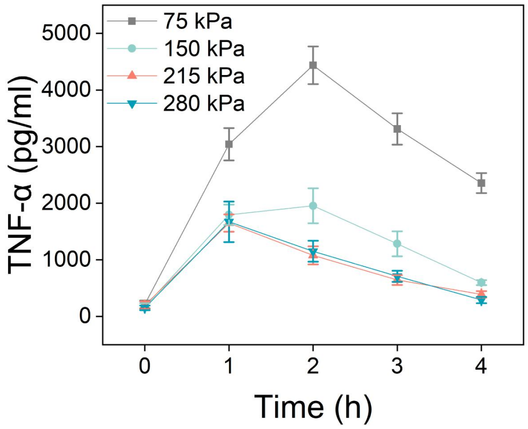

Fig. S28. Attenuation of LPS-induced systemic inflammation following NWUS stimulation at different acoustic pressures (n = 3 independent animals). It shows the effectiveness of NWUS stimulation in alleviating inflammation.

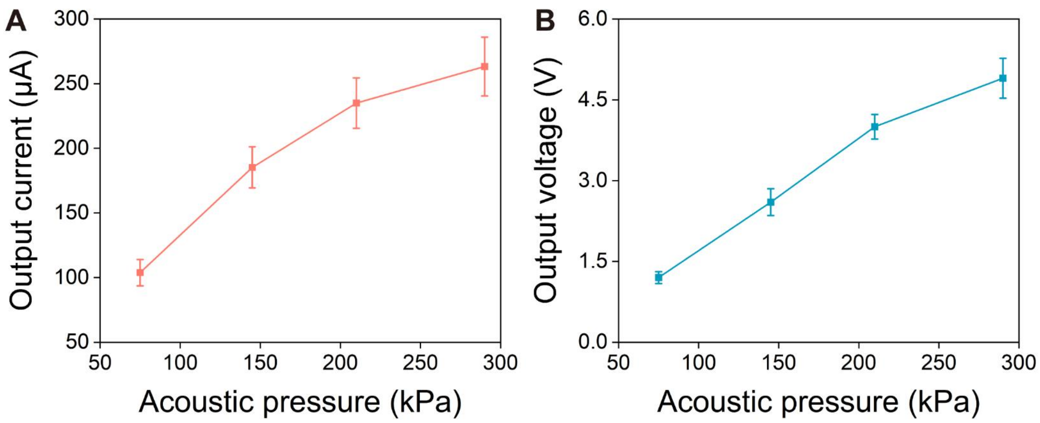

Fig. S29. Output performance of NWUS under *in vivo* vagus nerve loading conditions. (A) Output current measurements under the *in vivo* loading conditions. **(B)** Output voltage measurements under the *in vivo* loading conditions.

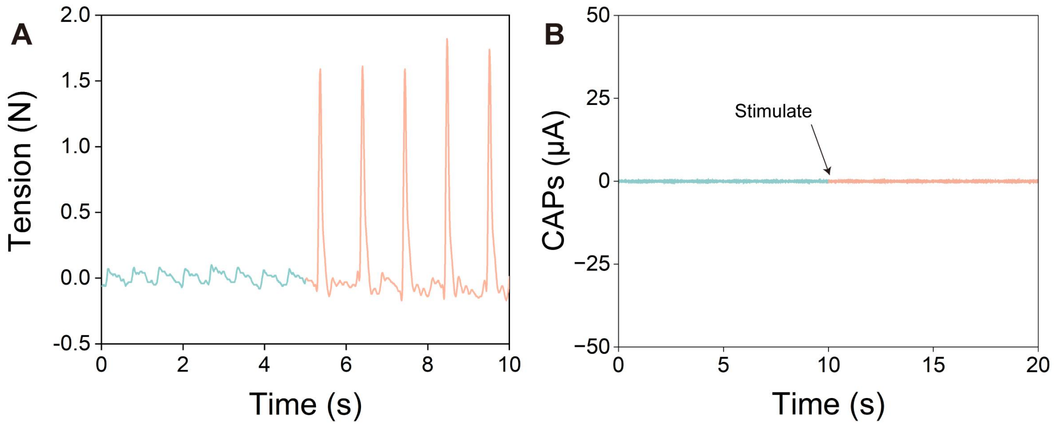

Fig. S30. Electrophysiological signal induced by NWUS-enabled stimulation and direct ultrasound stimulation under 150 kPa. (A) Gastrocnemius muscle tension induced by NWUS-enabled stimulation of the rat sciatic nerves. **(B)** Recording of electrophysiological signal induced by direct ultrasound stimulation under an acoustic pressure of 150 kPa.

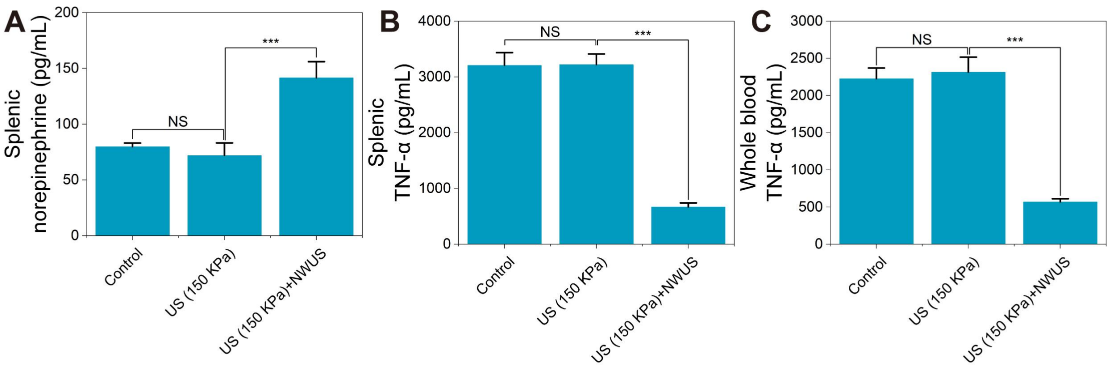

Fig. S31. Assessment of immune and neurochemical responses to ultrasound stimulation with or without NWUS. (A) Splenic norepinephrine (NE), **(B)** splenic TNF-α, and **(C)** whole blood TNF-α levels measured in control animals, animals receiving external ultrasound stimulation at 150 kPa applied to the cervical vagal region (US, 150 kPa), and animals receiving ultrasound stimulation in the presence of NWUS implantation (US, 150 kPa + NWUS).

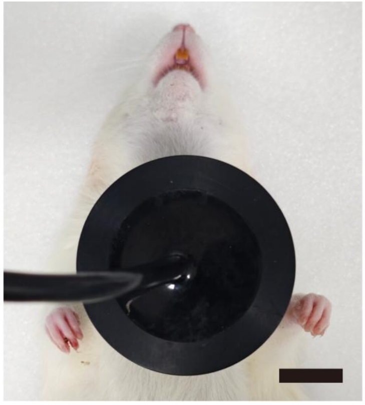

Fig. S32. Photographs of the *in vivo* ultrasound treatment setup. The low-intensity ultrasound transducer is placed in direct contact with the skin overlying the implantation site. Scale bar, 1cm.

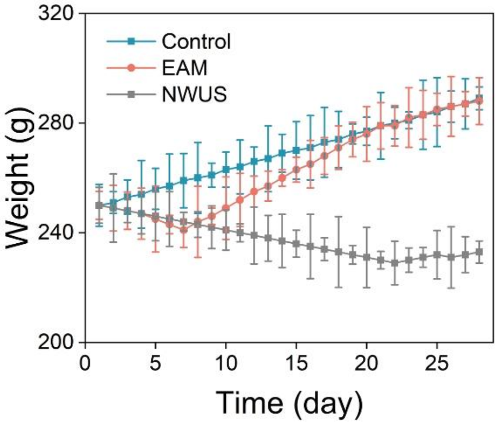

Fig. S33. Body weight change curves of rats over time (n = 5 independent animals). It shows progressive weight loss in the EAM group and recovery following NWUS-enabled VNS.

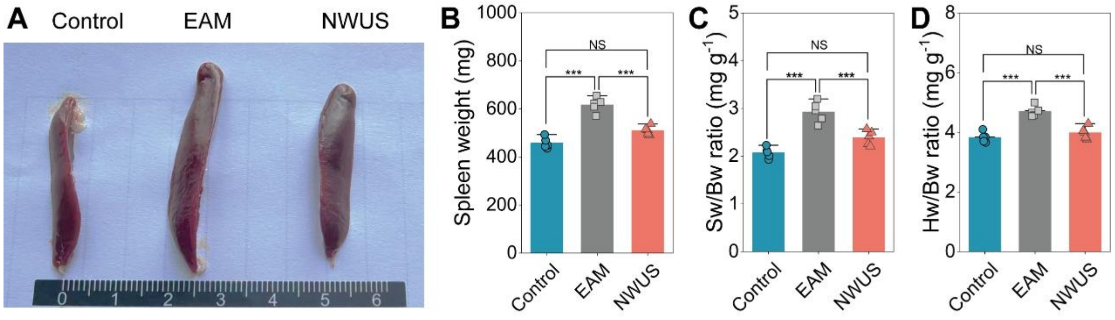

Fig. S34. Effect of NWUS treatment on spleen and heart-to-body weight ratios (n = 5 independent animals). (A) Representative images of spleens from each group. **(B)** Quantification of absolute spleen weights. **(C)** Ratios of spleen weight to body weight (Sw/Bw) across groups. **(D)** Ratios of heart weight to body weight (Hw/Bw) across groups. Data are presented as the mean ± SD in **(B-D)** and were analyzed by one-way ANOVA first, and then by the Tukey's post hoc test. ***$P \leq 0.001$. NS, not significant.

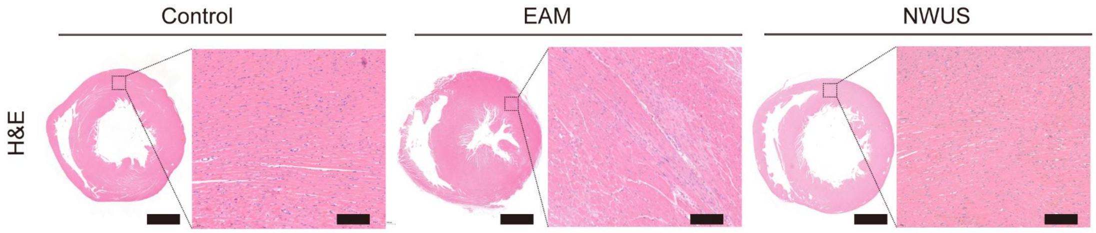

Fig. S35. Histological analysis of myocardial structure and inflammatory infiltration (images reused from Fig. 4G). Representative hematoxylin and eosin (H&E) staining of hearts in the control, EAM, and NWUS groups at day 28, confirming the reduction of diffuse inflammatory infiltrates following VNS treatment (these images display the corresponding H&E staining of the same tissue regions from the identical hearts shown with Masson's staining in Fig. 4G). Scale bar, 2 mm (left), 100 μm (right).

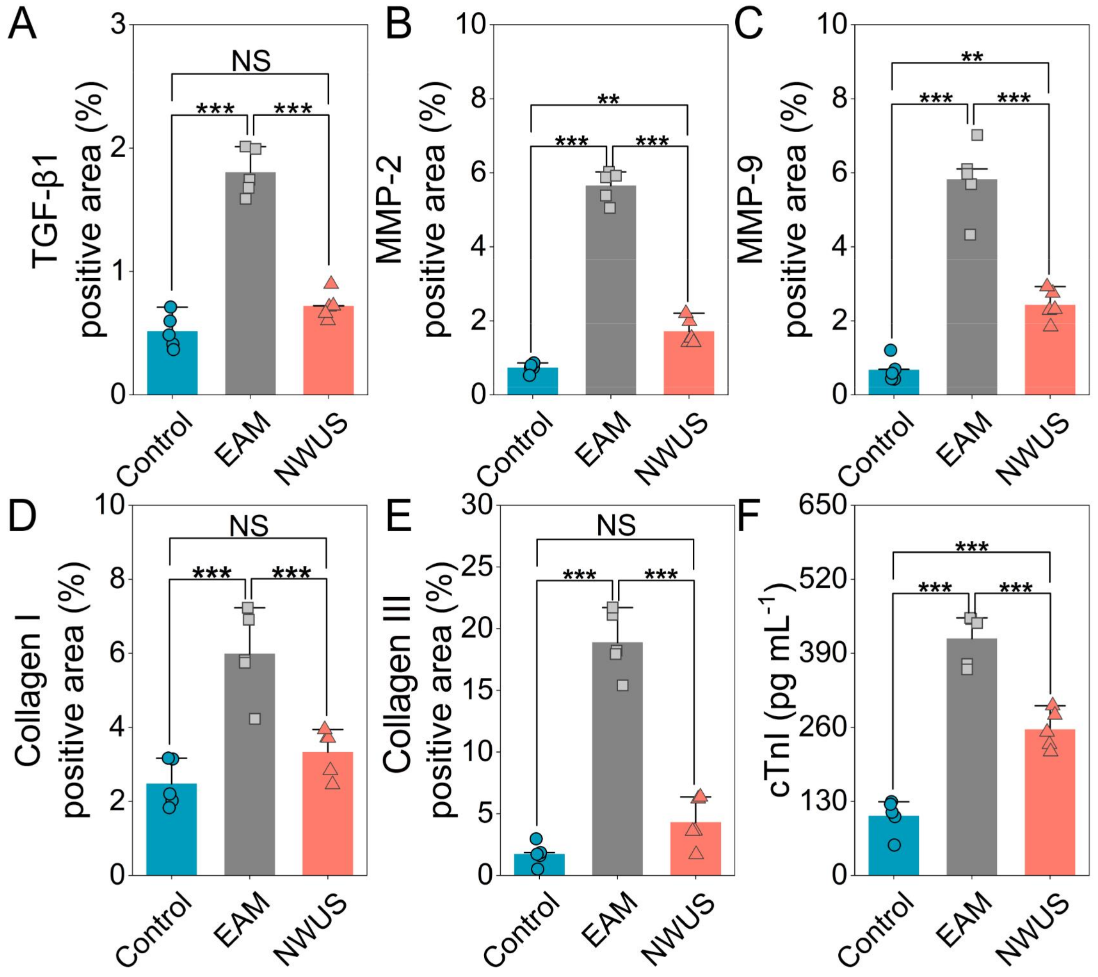

Fig. S36. Quantitative analysis of myocardial fibrotic markers. (A-E) Quantitative analysis of transforming growth factor β1 (TGF-β1) positive area (%), matrix metallopeptidase-2 (MMP-2) positive area (%), matrix metallopeptidase-9 (MMP-9) positive area (%), Collagen I positive area (%), and Collagen III positive area (%). **(F)** Quantification of serum levels of cardiac troponin I (cTnI) (n = 5 independent animals). Data are presented as the mean ± SD in **(A-F)** and were analyzed by one-way ANOVA first, and then by the Tukey's post hoc test. *$P \leq 0.05$, ***$P \leq 0.001$. NS, not significant.

Table S1. Comparison of wireless power transfer modalities for small-scale peripheral nerve interfaces.

	**This work**	**Inductive RF**	**Magnetoelectric (ME)**
**Thickness**	~0.079 mm	~0.5 mm *(28)*	~0.3 mm *(30)*
**Flexibility**	High	Medium	Low (intrinsic material stiffness)
**Alignment tolerance**	~90% transmission efficiency maintained under 5 mm misalignment	Efficiency degrades sharply with lateral and angular misalignment between coils *(29)*	Reported robust power delivery under several millimeters of translational offset *(30)*
**Impedance matching**	No impedance-matching network required	Implant coil must be resonantly tuned using matching network	No separate coil required
**Fixation**	Conformal self-wrapping interface enables intimate nerve contact	Requires secondary fixation	Requires secondary fixation
**Implantation safety**	Integrated implantation avoids stress-induced injury and electrode displacement	Coil displacement relative to transmitter degrades coupling and may introduce lead strain	Tensile forces exerted by lead wires may induce nerve injury
